# Dual effect of TAT functionalized DHAH lipid nanoparticles with neurotrophic factors in human BBB and microglia cultures

**DOI:** 10.1186/s12987-022-00315-1

**Published:** 2022-03-17

**Authors:** Sara Hernando, Polyxeni Nikolakopoulou, Dimitrios Voulgaris, Rosa Maria Hernandez, Manoli Igartua, Anna Herland

**Affiliations:** 1 Center for the Advancement of Integrated Medical and Engineering Sciences (AIMES), Karolinska Institutet and KTH Royal Institute of Technology, 171 77 Stockholm, Sweden; 2 Department of Neuroscience, Karolinska Institutet, 171 77 Stockholm, Sweden; 3 Division of Nanobiotechnology, Department of Protein Science, Science for Life Laboratory, KTH Royal Institute of Technology, Solna, Sweden; 4 Division of Micro and Nanosystems, KTH Royal Institute of Technology, 171 77 Stockholm, Sweden; 5 NanoBioCel Research Group, Laboratory of Pharmaceutics, School of Pharmacy, University of the Basque Country (UPV/EHU), 01006 Vitoria-Gasteiz, Spain; 6 Biomedical Research Networking Centre in Bioengineering, Biomaterials and Nanomedicine (CIBER-BBN), Institute of Health Carlos III, 28029 Madrid, Spain; 7 Bioaraba, NanoBioCel Research Group, 01006 Vitoria-Gasteiz, Spain

**Keywords:** Blood–brain barrier, BMECs, DHA, HMC3 microglia cell line, Neuroinflammation, iPS cells, Drug delivery, Neurodegenerative disease, Nanoparticles

## Abstract

**Background:**

Neurodegenerative diseases (NDs) are an accelerating global health problem. Nevertheless, the stronghold of the brain- the blood–brain barrier (BBB) prevents drug penetrance and dwindles effective treatments. Therefore, it is crucial to identify Trojan horse-like drug carriers that can effectively cross the blood–brain barrier and reach the brain tissue. We have previously developed polyunsaturated fatty acids (PUFA)-based nanostructured lipid carriers (NLC), namely DHAH-NLC. These carriers are modulated with BBB-permeating compounds such as chitosan (CS) and trans-activating transcriptional activator (TAT) from HIV-1 that can entrap neurotrophic factors (NTF) serving as nanocarriers for NDs treatment. Moreover, microglia are suggested as a key causative factor of the undergoing neuroinflammation of NDs. In this work, we used in vitro models to investigate whether DHAH-NLCs can enter the brain via the BBB and investigate the therapeutic effect of NTF-containing DHAH-NLC and DHAH-NLC itself on lipopolysaccharide-challenged microglia.

**Methods:**

We employed human induced pluripotent stem cell-derived brain microvascular endothelial cells (BMECs) to capitalize on the in vivo-like TEER of this BBB model and quantitatively assessed the permeability of DHAH-NLCs. We also used the HMC3 microglia cell line to assess the therapeutic effect of NTF-containing DHAH-NLC upon LPS challenge.

**Results:**

TAT-functionalized DHAH-NLCs successfully crossed the in vitro BBB model, which exhibited high transendothelial electrical resistance (TEER) values (≈3000 Ω*cm^2^). Specifically, the TAT-functionalized DHAH-NLCs showed a permeability of up to 0.4% of the dose. Furthermore, using human microglia (HMC3), we demonstrate that DHAH-NLCs successfully counteracted the inflammatory response in our cultures after LPS challenge. Moreover, the encapsulation of glial cell-derived neurotrophic factor (GNDF)-containing DHAH-NLCs (DHAH-NLC-GNDF) activated the Nrf2/HO-1 pathway, suggesting the triggering of the endogenous anti-oxidative system present in microglia**.**

**Conclusions:**

Overall, this work shows that the TAT-functionalized DHAH-NLCs can cross the BBB, modulate immune responses, and serve as cargo carriers for growth factors; thus, constituting an attractive and promising novel drug delivery approach for the transport of therapeutics through the BBB into the brain.

**Supplementary Information:**

The online version contains supplementary material available at 10.1186/s12987-022-00315-1.

## Background

Human brain microvascular endothelial cells (BMECs) form a tight barrier—the blood–brain barrier (BBB)—, which shows high selectivity and low transcellular and paracellular transport [1, 2]. BBB breakdown allows the influx of neurotoxic blood-derived agents, cells, and pathogens into the brain parenchyma, and initiates a cascade of inflammatory and immune responses in the neural tissue, followed by the activation of several neurodegenerative mechanisms [3]. Neurodegenerative diseases (NDs) such as Alzheimer’s disease (AD) and Parkinson’s disease (PD) have escalated in prevalence; in fact, World Health Organization (WHO) forecasts that, in the years to come and as life expectancy increases, the number of patients suffering from NDs will increase considerably [4–6]. Neurodegeneration manifests itself with functional deterioration and ultimate loss of neurons; however, the molecular cues governing disease initiation and progression remain elusive. Furthermore, up to today, the existing treatments only manage the symptoms without slowing down disease progression. Thus, the ongoing neurodegenerative process remains untreated [7, 8].

Drug delivery to the human CNS remains one of the biggest challenges for targeted therapy development. Effective agents should have a multifaceted character enabling them to surpass the innate selectivity of the human BBB and treat the respective disease and its symptoms. The clinical picture among the various NDs differs vastly. Nevertheless, they share some common characteristics of neurodegeneration, such as neuronal loss, insoluble proteins deposits, oxidative stress, mitochondrial dysfunction, and neuroinflammation [9–11]. The sustained neuroinflammation disrupts the balance between neurotrophic and neurotoxic factors. Thus, growth factors (GFs) have emerged as putative therapeutic candidates. GFs exhibit a neuroprotective character: they act on growth, proliferation, and differentiation while regulating neuroinflammation, thus promoting endogenous brain repair [12]. Among them, glial cell-derived neurotrophic factor (GDNF) and vascular endothelial factor (VEGF) have emerged as some of the best candidates for PD and AD, respectively [13–15]. More recently, polyunsaturated fatty acids (PUFAs) and especially omega (n)-3, have gained attention as functional lipids; recent data support the beneficial role of these natural compounds for the prevention and/or treatment of NDs due to their anti-inflammatory, antioxidative, and neuroprotective properties [16–18]. However, most of these novel modalities cannot cross the blood–brain barrier (BBB) and require an invasive administration route such as intrathecal or intracerebroventricular [19].

Nanotechnology-based drug delivery systems (DDSs) comprise an alternative approach for brain targeting [20, 21]. Nanostructured lipid carriers (NLCs), in particular, have gained popularity due to their ability to entrap highly lipophilic drugs and proteins, protect them from degradation, and enhance their stability [22–24]. Until recently, the lipids used for NLC formation were inert excipient without any active role in preventing or treating the symptomatology of the disease [25]. One example is Miglyol, a component of NLCs that we have evaluated clinically [26–28]. Lately, however, functional lipids such as oleic acid have been proposed as components of the lipid matrix of NLCs [29–31]. Docosahexaenoic acid (DHA) is one of the most abundant PUFAs in the brain and, among other functions, regulates cell survival, neuroinflammation, and BBB permeability [32]. Following a similar approach with the previously mentioned reports, we have recently developed a new functional nanocarrier with the hydroxylated derivate of docosahexaenoic acid (DHAH), so that the nanoparticles (NPs) themselves could exhibit neuroprotective and anti-inflammatory effects (DHAH-NLCs) [33]. Moreover, the functionalization of particles after surface modification leads to increased penetrance via the BBB [34]. For example, previous studies with chitosan (CS) and cationic cell-penetrating TAT peptide- trans-activating transcriptional activator from HIV-1-coated particles showed enhanced barrier permeability and led to increased drug bioavailability in various brain regions in mice [27, 35, 36]. Human-oriented models are essential to assess the potential of these particles to cross the human BBB and potentially treat brain pathologies.

Availability of primary BBB tissue is sparse, often derived from postmortem human brains or residuals of biopsies; thus, primary BMECs lose their barrier properties rapidly during cell culture [37, 38]. While endothelial cell lines offer an alternative to circumvent availability and reproducibility issues, they fail to form tight junctions and show low transepithelial/endothelial electrical resistance (TEER) with values ranging from 2 to 50 Ω x cm^2^ [38–40]. Animal models are the gold standard in CNS research and drug discovery; nevertheless, rodent studies suffer from interspecies differences and low relevance to humans. Thus, current CNS disease modeling suffers from low translatability to the clinic, as shown by the failure of drugs that reach the clinical trials [41–45]. Human induced pluripotent stem cell (hiPSC)-derived BMECs recapitulate many of the physiological properties of the brain endothelium, including tight, organized junctions, functional efflux transporters, and high TEER values, thereby providing an ideal platform for drug testing [37, 46–48]. Hence, hiPSC-based BMECs can serve as novel culture systems to evaluate novel drugs’ efficacy in CNS diseases.

Microglia constitute 5–12% of the cells in the CNS. They are the principal resident immune cells of the brain and are involved, among other physiological functions in neuroinflammation [49–51]. Neuroinflammation is a defense mechanism that protects the brain tissue from various insults. Nonetheless, a common feature of NDs is tissue damage due to aberrant microglial activation and sustained neuroinflammation. Thus, a key element of effective therapeutics for NDs is their potential to regulate microglial responses. To this end, several studies identified molecules that are putative modulators of the neuroinflammatory state. Indeed, the use of molecules—such as the previously mentioned PUFAs, GDNF, or VEGF—with anti-inflammatory and antioxidative properties have emerged as a feasible therapeutic option for NDs. These agents could modulate microglial activation and counteract neuroinflammation in paradigms of AD and PD [52–54]. Hence, modulating microglial responses is crucial to develop effective therapeutics for the human CNS; such agents must have properties that enable them to (a) reach the brain tissue and (b) treat the respective NDs. However, this is challenging due to the BBB, which hinders entrance to the brain tissue via the vasculature. Nanotechnology-inspired DDSs emerged as a feasible solution for brain targeting; these novel nanoparticles can modulate neuroinflammatory processes and counteract ND progression once they bypass the BBB [20, 27].

In summary, this study serves a dual scope. First, using hiPSC-derived BMECs, we investigated the ability of newly generated, TAT-functionalized, DHAH-NLCs to cross the BBB. Next, we tested if our modified NPs could regulate inflammatory responses in human microglia. Moreover, we investigated if the encapsulation of VEGF or GDNF in our DHAH-NLCs would further enhance our particles’ neuroprotective and anti-inflammatory abilities.

## Methods

### Materials

The references of the main products used in this work are summarized in Additional file 1: File 2.

### Nanostructured lipid carriers (NLCs) preparation

**Fig. 1:**
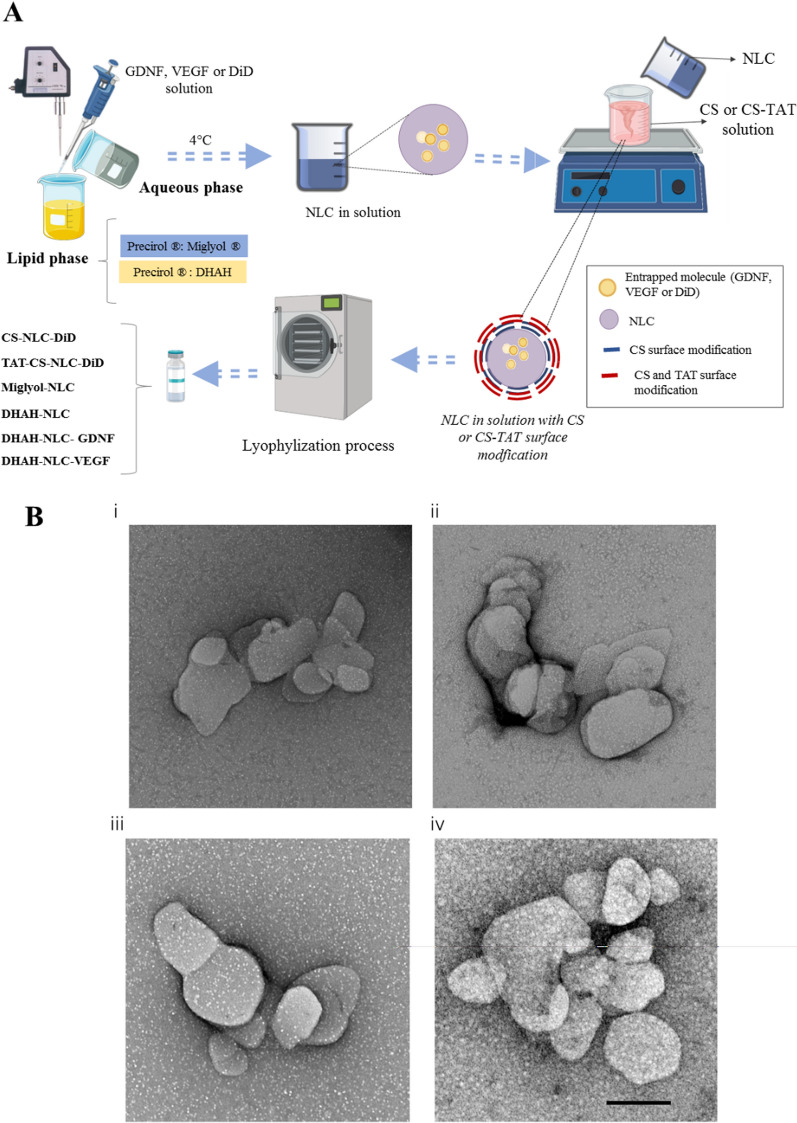
NLC preparation and characterization images. **A** Schematic representation of the different steps followed to NLC preparation **(**This figure was created using Servier Medical Art templates, licensed under a Creative Commons Attribution 3.0 Unported License; https://smart.servier.com) **B** TEM images of the NLCs. (i) Miglyol-NLC (ii) DHAH-NLC (iii) DHAH-NLC-GDNF (iv) DHAH-NLC-VEGF. Scale bar 200 nm

NLCs were prepared using a previously published melt-emulsification technique [27, 55] (Fig. 1A). Firstly, a mixture of solid and liquid lipids (Precirol ATO ®5 1.75%, w/v and Miglyol or DHAH 1% w/v) was melted 5 °C above their melting point (56 °C). Then, an aqueous solution containing Tween 80 (3%, w/v) and Poloxamer 188 (2%, w/v) was heated at the same temperature and added to the lipid phase under continuous stirring, for 60 s, at 50 W (Bradson Sonifier 250). The resulted emulsion was maintained under magnetic stirring for 15 min at room temperature (RT) and immediately cooled at 4–8 °C overnight to obtain the NLCs after lipid solidification.

NLCs surface was modified with CS only or with both CS and TAT. The CS: TAT employed ratio was 1:0.01 (w/w). For the surface modification with CS, the NP dispersion was added dropwise to an equal volume (4 ml) of a CS solution (0.5%, w/v) under continuous agitation at RT for 20 min. After the coating process, the NLC dispersion was centrifuged in Amicon filters (Amicon, “Ultracel-100 k”, Millipore, USA) at 2,500 rpm (MIXTASEL, P Selecta, Spain) for 15 min, washed three times with Milli Q water and lyophilized for 42 h (LyoBeta 15, Telstar, Spain). TAT-peptide was covalently linked to the surface of CS coated NLCs by a surface activation method previously described by our research group [55, 56]. Briefly, 250 µl of EDC (1-ethyl-3-(3-dimethylaminopropyl) carbodiimide hydrochloride) in solution (1 mg/ml) and 250 µl of sulfo NHS (*N*-Hydroxysulfosuccinimide) in 0.02 M PBS (1 mg/ml) were added dropwise to a 4 ml CS solution (0.5% w/v, in PBS 0.02 M), under magnetic stirring (2 h, RT). For the coupling of TAT, 250 µl of the TAT solution (1 mg/ml) in PBS (0.02 M; 7.4 pH) was added dropwise to the activated CS under gentle agitation. The TAT-CS solution was maintained under stirring for another 4 h at RT and then incubated at 4 °C overnight. The day after, the NLCs were coated with TAT-CS; NLC dispersion previously prepared was added dropwise to the TAT-CS solution under continuous agitation for 20 min at RT. After the coating process, TAT-CS-NLC nanoformulation was centrifuged and lyophilized, as described in the previous paragraph.

Finally, the neurotrophic factors; glial cell-derived neurotrophic factor (GDNF) or vascular endothelial growth factor (VEGF), were loaded in the TAT-CS-NLCs previously developed formulation at a concentration of 0.125% (w/w) (DHAH-NLC GNDF and DHAH-NLC-VEGF) to probe the therapeutic effect of these nanoformulations in our microglial cultures after LPS *stimuli*. For the BBB transport assays, the lipophilic dye DiD was incorporated into the NLC (TAT-CS-NLC-DiD and CS-NLC-DiD) at a concentration of 0.5% (w/w). We developed these formulations following the previously described protocol with slight modifications; here, we included the relevant GF or dye, depending on the desired formulation, in the lipid phase, before the sonication process (Fig. 1A). Table 1 summarizes the six different formulations-regarding the used lipid, the entrapped molecule, or the coating process with CS and TAT—that we developed and utilized in this study.

**Table 1:** Composition of the different NLCs used in the study

Formulation name	Liquid lipid	Surface modification	Entrapped molecule (%) w/w	Cell model to perform functional assays
CS-NLC-DiD	DHAH	CS	DiD (0.5)	hiPSCs derived BMECs
TAT-CS-NLC-DiD	DHAH	CS and TAT	DiD (0.5)	hiPSCs derived BMECs
Miglyol-NLC	Miglyol	CS and TAT	-	Microglial cell line—HMC3
DHAH-NLC	DHAH	CS and TAT	–	Microglial cell line—HMC3
DHAH-NLC-GDNF	DHAH	CS and TAT	GDNF (0.125)	Microglial cell line—HMC3
DHAH-NLC-VEGF	DHAH	CS and TAT	VEFG (0.125)	Microglial cell line—HMC3

### NLC characterization: particle size, zeta potential, morphology, and encapsulation efficiency

The mean particle size (Z-average diameter), the polydispersity index (PDI), and the zeta potential were measured by Dynamic Light Scattering (DLS), through Laser Doppler micro-electrophoresis (Malvern Zetasizer Nano ZS, Model Zen 3600; Malvern Instruments Ltd, UK). For each formulation, we performed three replicate analyses. The data are expressed as the mean ± SEM. To investigate the morphology of the NPs we performed transmission electron microscopy (TEM) with a JEOL JEM 1400 Plus.

The encapsulation efficiency (EE) of the NLCs was determined by an indirect method, in which we measured the non-encapsulated GDNF, or VEGF presented in the supernatant obtained after the filtration/centrifugation process described in the previous section. The EE (%) of the GF was determined by the ELISA technique using the following equation:

$$\mathrm{EE }(\mathrm{\%}) =\frac{total \,GF\, content-free\, amount\, of\, GF}{total\, GF\,content}\times 100$$

The absence of DiD release from NLCs in transport buffer was assessed by our group previously [28, 55]; thus, it is not described in the present work.

### hiPSC differentiation to BMECs

#### hiPSCs maintenance

The hiPSC line used in this study was the Control 9, and it was obtained from the iPS Core at Karolinska Institutet [57]. hiPSCs were cultured in Matrigel-coated (0.5 mg/6 well plate) six-well plates with mTeSR^TM^1 medium. When the confluence of *hiPSCs* was up to 80%, cells were passaged after 5 min incubation at 37 °C with Versene solution. Then, using a 5 ml pipette, cells were gently dissociated and passaged 1:3–1:8 split ratios onto Matrigel-coated six-well plates with mTeSR^TM^1 medium. For BMEC differentiation, we used hiPSCs up to passage 41, being in the typical passage range for BMEC differentiation [58].

#### BBB development

For BBB differentiation, we followed the protocol by Neal et al*.,* with slight modifications [47]. hiPSCs were maintained in mTeSR media as described above. One day before differentiation induction (D-1), cells were washed with Dulbecco's phosphate-buffered saline (DPBS); 500 µl of TrypLE was added to each well and passaged after 5 min incubation at 37 °C. Next, cells were diluted 1:5 in mTeSR media, centrifuged for 3 min at 200 rcf, and resuspended in mTeSR media supplemented with ROCK inhibitor (10 µM). HiPSCs were seeded onto Matrigel-coated six-well plates at 16 K/cm^2^ cell density. The day after (D0), mTeSR was removed and changed to E6 media. We repeated the procedure daily for four days (D0-D3). Then, media was switched to Human Endothelial Serum Free Media (hESFM media) supplemented with 1X B27, 20 ng/ml bFGF and 10 µM RA termed hESFM complete media. Cells were maintained in this media for two consecutive days without a media change. After those two days, the media was removed, wells were washed with DPBS and incubated with TrypLE for 20 min to 30 min at 37 °C until a single cell suspension was formed. The cells were then subcultured onto 6.5 mm Transwell filters with 0.4 µm pore size, coated with a mixture of 400 μg/mL collagen IV and 100 μg/mL fibronectin in water; 3.3 × 10^5^ cells were seeded in each Transwell with hESFM complete media.

24 h after subculture, TEER was measured using STX2 chopstick electrodes and an EVOM2 voltohmmeter (World Precision Instruments). Media was then switched to hESFM with B27 without bFGF and RA. The next day (48 h after subculture), TEER was measured, and functional assays were performed.

#### BBB assessment: TEER measurement

All experiments in this study were performed with the hiPSC cell line, Control 9. TEER values show the mean of three independent differentiations (n = 3), while in each differentiation, three to four Transwells were utilized as technical replicates. TEER was measured 24 h, and 48 h post subculture and all values were corrected for the resistance of an empty, coated Transwell filter. Only cell monolayers with TEER above 2000 Ω x cm^2^ at 48 h post- subculture were selected for follow-up experiments.

#### Immunocytochemistry

Transwell inserts with BMECs were washed twice with DPBS and incubated with 4% paraformaldehyde for 20 min. Cells were then washed three times with DPBS for a minimum of 5 min per wash. The fixed cells were blocked and permeabilized for a minimum of 1 h at RT in DPBS with 10% goat serum and 0.1% Triton X-100 in DPBS. After three washes, cells were then incubated with ZO-1 primary antibody (1:100) in staining solution containing 1% goat serum and 0.01% Triton X-100 in DPBS overnight at 4 °C. The following day, cells were washed 3 times and incubated with the secondary antibody, an anti-Mouse IgG1 (γ1), CF™488A antibody (1:1000) in staining solution for 1 h at RT. After three rinsing steps, the cells were incubated with 300 nM of 4′,6-diamidino-2-pheny-lindoldihydrochloride (DAPI) for 10 min to label the nuclei. Inserts were then washed twice with DPBS, cut, and mounted with ProLong Glass Antifade Mountant on glass slides. Cells were visualized using a Zeiss laser scanning confocal microscope (LSM 710) using a 25X and a 40X objective.

#### NLC transport across BMECs differentiated from hiPSC

Transport of NLCs across BMECs was studied quantitatively by fluorescence measurement (Plate Reader Infinite M1000, Tecan, Switzerland) and qualitatively by confocal laser scanning microscopy (LSM 710), using DiD (λ _em_ = 644 nm, λ _ex_ = 665 nm) with labeled NLCs. Here, two different formulations described in Table 1 were used, the CS-NLC-DiD and the TAT-CS-NLC-DiD. hiPSC-derived BMECs were seeded on Transwell filters as described above (3.3 × 10^5^ cells per insert), and the transport of the different NLCs was evaluated 48 h after. Our previous experiments confirm barrier integrity for 4 h when treated with 1 mg/ml NLCs (Additional file 1: File 5). Thus, Transwells with TEER values above 2000 Ωxcm^2^ were selected for the transport studies, and BMCEs were treated with 1 mg/ml of the NLCs for 2 h in total. At the beginning of the assay (t = 0), half medium was removed from the donor chamber, and fresh medium (hESFM with B27) containing 2 mg/ml NLC was added. At different time points (0 min, 30 min, 60 min, 90 min and 120 min), 50 µl of volume sample was collected from the basolateral side, and 50 µl of fresh media was added to the same chamber. Like above, cells were placed on an orbital shaker at 37° throughout the assay. The NLC concentration was determined by fluorescence measurement (Plate Reader Infinite M1000, Tecan, Switzerland). The relative fluorescent signal was correlated to a standard linear curve (0–125 µg/ml in serial dilutions). The transport rate of NLC is expressed as the percentage of the NLC transported mass (of the dose in the donor chamber). After transport experiments, the supernatant was removed, and cell monolayers were fixed in PFA 4% for subsequent staining as described previously. The NLCs that were not entrapped into BMECs were removed during washing and staining process. Images were captured using a Zeiss confocal microscope (LSM710).

### HMC3 microglia cell culture

#### HMC3 cell line viability assay

HMC3 cells were maintained in DMEM/F12 medium containing 10% FBS under standardized conditions (95% relative humidity, 5% CO_2_, 37 °C). To assess the working concentration for the NLCs Alamar Blue assay was carried out. Cells were seeded at 10 K/cm^2^ in a 96-well plate for 24 h to allow cell attachment. The day after, different doses of NLC formulations described in Table 1 were added (DHAH-NLC, Miglyol-NLC, DHAH-NLC-GDNF and DHAH-NLC-VEGF). The concentration for DHAH-NLCs refers to DHAH functional lipid concentration in µM: 12.5, 25, 50 and 100 (Additional file 1: File 3). To check the differences in cell viability and functional assays between DHAH functional lipid and Miglyol lipid [33], Miglyol-NLCs (equivalent to 15.5, 31, 62,124 µg/ml NPs concentration), was used as an internal control (M1–M4). Miglyol is a well-known excipient previously used and clinically evaluated by our research group [26–28]. For the two different tested GF: GDNF and VEGF (DAH-NLC-GNDNF) and (DHAH-NLC-VEGF) 12.5, 25, 50, and 100 µM for DHAH lipid content and 12.5, 25, 50 and 100 ng/ml for the GF concentration were used (Additional file 1: File 3). The different formulations and doses were incubated with HMC3 for 24 h and 48 h. Afterwards, the viability was assessed using the AlamarBlue assay, following the manufacturer’s protocol. Briefly, 10 µL (1:10 in cell culture media) of the alamarBlue cell viability reagent was added to the cells. After 4 h incubation, the absorbance of the mixture was read at 570 nm, using 600 nm as reference wavelength (Plate Reader Infinite M1000, Tecan, Switzerland). The absorbance was directly proportional to the number of living cells in the culture. Cell viability for each condition is expressed as the percentage of the negative control (Control−), which received no treatment and was set as 100%. For positive control (Control +), cells were treated with DMSO 10% for at least 24 h. The procedure was done following INTERNATIONAL STANDARD ISO 10993-5 for Biological evaluation of medical devices [59]. Therefore, the nanoparticles were considered non-cytotoxic when the highest concentration of the sample extract was ≥ 70% of the control group.

#### HMC3 activation with LPS

To test the efficacy of our NPs in modulating neuroinflammation, we treated our microglial cultures with LPS (100 ng/ml, InvivoGen). Cells were seeded at 10 K/cm^2^ or 15 K/cm^2^, depending on the desired downstream analysis strategy; to investigate the genes involved in the neuroinflammatory process, we performed gene expression analysis with RT-qPCR (seeding density 15 K/cm^2^), whereas to assess cytokine responses we performed Multiplex assay (seeding density 10 K/cm^2^). Our experimental design consisted of two experimental conditions (Additional file 1: File 6); in the preconditioning assay, we tested if the NLCs affected our microglia, while in the anti-inflammatory assay, we tested if they could modulate inflammation following LPS treatment. The working concentration of NLCs was determined with the AlamarBlue assay as described in the previous section.

##### Preconditioning assay: test for effects of the NLCs on microglia

We tested the following formulations: DHAH-NLC (25 µM of DHAH lipid), DHAH-NLC-GDNF (25 µM of DHAH lipid and 25 ng/ml GDNF), DHAH -NLC-VEGF (25 µM of DHAH lipid and 25 ng/ml VEGF) and finally, Miglyol-NLC—an equal dose of NLCs was used as internal control, 31 µg/ml, (M2); to show the differences between formulating with Miglyol or DHAH functional lipid. Here, cells were treated with the NLCs 24 h after seeding and samples were collected the next day for downstream analysis. As positive control (LPS treated cells; Control ^+^), we used cells that we treated with 100 ng/ml LPS for 24 h, whereas as negative control (media change; Control ^–^), we used cells that we performed only media change.

##### Anti-inflammatory assay: testing for effects of the NLCs on inflamed microglia

At this point, our cells were pre-treated with the various NLCs for 24 h, when they were challenged with LPS. Following LPS treatment, cells were treated again with the respective NLCs (same dose as in preconditioning assay), and samples were collected 24 h after for downstream analysis. Similar to above, as a positive control (LPS treated cells; Control ^+^), we used cells that we treated with LPS for another 24 h, whereas as negative control (media change; Control ^–^), we used cells that we performed only media change.

For both conditions, we analyzed six groups (Additional file 1: File 6).

#### RT-qPCR

Total RNA isolation and purification was performed using the High pure RNA isolation Kit from Roche, following the manufacturer’s instructions. Next, RNA quantity and quality were assessed by NanoDrop 1000 Spectrophotometer (Thermo Scientific). 500 ng RNA was used for cDNA synthesis using High-Capacity RNA-to-cDNA Kit according to the manufacturer’s instructions. TaqMan-based qPCR assay was used to perform gene expression analysis using a QuantStudio 5 Flex Real-Time PCR System. TaqMan Assay gene names (assay ID) are summarized in Additional file 1: File 4. Samples were run in duplicates in 96-well plates. Relative gene expression was evaluated with the ΔΔCt method after normalization to glyceraldehyde 3-phosphate dehydrogenase (GAPDH). All the experiments were run in triplicates.

#### Multiplex assay

Cytokine levels of cell culture supernatant were measured on the U-Plex MSD electrochemiluminescence multi-spot assay platform (MesoScale Diagnostics, Rockville USA). Concentration ranges 2,000 to 0.33 pg/ml for IL-6, 2,200 to 0.15 pg/ml for IL-8, 3, 700 to 0.14 pg/ml for IL-10, 3, 700 to 0.51 pg/ml for TNF-α, 3, 800 to 0.15 pg/ml for IL-1β and 17,000 to 1.7 pg/ml for IFN- γ were used. Cell numbers in the preconditioning and anti-inflammatory conditions were measured after DAPI staining (300 nM) with ImageXpress Pico Automated Cell Imaging System. No differences in HMC3 cell viability were observed after LPS and/or NLCs incubation (p > 0.05, One-way Anova) (Additional file 1: File 7). Thus, data are represented as pg/ml and have not been normalized to cell numbers. Cell viability for each condition (treated cells) is expressed as the percentage of living cells compared to the nontreated cells (media change; Control ^–^), which was set as 100%. MSD assay was performed according to the manufacturer’s instructions. MSD testing was conducted in a single laboratory by a single technician at the SciLifeLab (Stockholm, Sweden). All the experiments were run in triplicate.

#### Statistical analysis

All results are expressed as means ± SEM unless otherwise stated. For hiPSC-derived BMECs, the TEER value shows the mean of three independent differentiation experiments, while in each differentiation, three to four Transwells served as technical replicates. Two-way ANOVA followed by Bonferroni's posthoc test (p < 0.05) was used to analyze the data. In the permeability assay analysis, we performed one-sample t-test analysis. In gene expression and cytokine release analyses, data is expressed as mean ± SEM of three independent experiments. One-way ANOVA followed by Bonferroni's posthoc test (p < 0.05) was used. The data was analyzed comparing the mean of each group to the mean of Control^–^. Experimental data were analysed using GraphPad Prism (v. 6.01, GraphPad Software, Inc.). The Multiple comparisons between all the groups –the pair-wise test-are presented in Additional file 1: Files 9 and 10, where One-way ANOVA followed by Turkey’s posthoc test with p < 0.05 was performed. For the gene expression analysis, statistics were performed using the delta-Ct values. P *values* < 0.05 were considered significant.

## Results

### Nanoparticle characterization

We developed several novel nanoformulations for this study, which vary in the surface coating, the used lipid, or the encapsulated molecule (Fig. 1A). Depending on the downstream experiments, our NLCs encapsulated GFs (GDNF or VEGF) or the fluorescent tracer DiD. Table 2 summarizes the mean particle size, polydispersity index (PDI), zeta potential and EE, for the GF-entrapping NLCs. Since, in our previous studies, we could not detect any release of the DiD tracer from NLCs, we assumed that the EE is 100% [28, 55]. As shown in Table 2, all formulations were uniform in size (100-200 nm) and had pDI values below 0.5, indicating a homogenous suspension. Moreover, they all exhibited positive zeta values, indicating that the CS and TAT coating process was successful. EE was around 85% for both GDNF and VEGF examined here. We utilized TEM to investigate the external morphology of our particles; our images show that our NPs showed uniform size without abnormalities (Fig. 1B i–iv).

**Table 2:** Physicochemical characterization of NLC used in all the experimental studies (one batch)

Formulation	Mean size after Lyophi. (nm)	PDI	Zeta potential (mV)	EE (%)
CS-NLC-DiD^*^				≈100
TAT-CS-NLC-DiD^(*)^				≈100
Miglyol-NLC	119.1 ± 18.0	0.323 ± 0.039	17.4 ± 0.8	–
DHAH-NLC	105.4 ± 25.6	0.400 ± 0.032	20.9 ± 0.5	–
DHAH-NLC-GDNF	257.1 ± 3.5	0.338 ± 0.022	18.0 ± 0.4	82.01 ± 1.67
DHAH-NLC-VEGF	264.1 ± 16.0	0.471 ± 0.076	20.5 ± 0.8	88.74 ± 0.37

^*^The presence of a fluorescent dye (DiD) makes it impossible to measure accurately the size, PDI and zeta potential of these two formulations

### Transport of the NLCs across the hiPSC-derived BBB

**Fig. 2:**
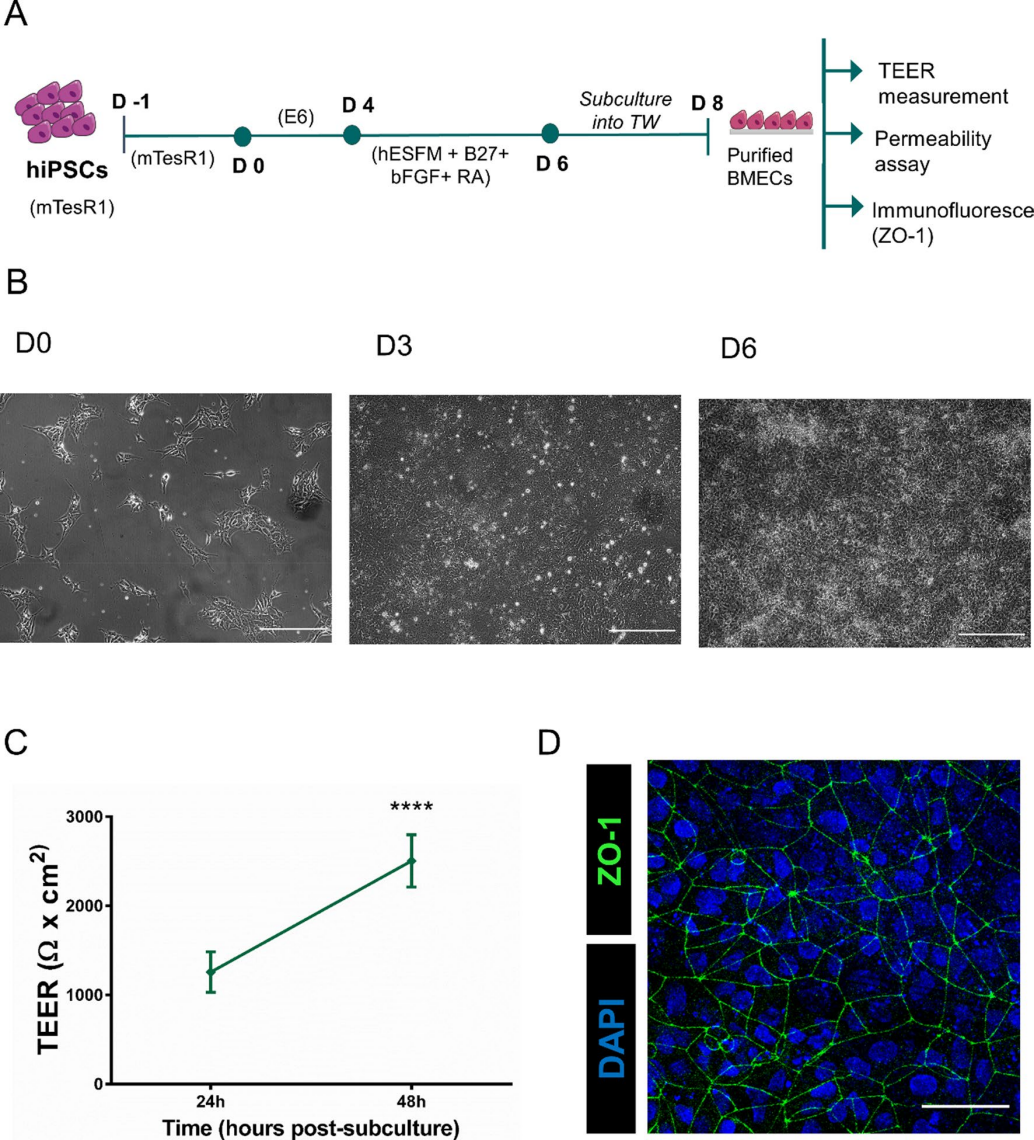
hiPSC-derived BBB differentiation **A** Scheme of the differentiation. (This figure was created using Servier Medical Art templates, licensed under a Creative Commons Attribution 3.0 Unported License; https://smart.servier.com). **B** Bright-field images of BMECs differentiation at different time points. (D0: after seeding, D3: complete coverage of the well-plate and D6: before subculturing onto TWs). Scale bar 200 µM. **C** TEER values after subculture onto Transwells at two different time points, 24 h and 48 h (Data are means ± SEM of three independent experiments. ^****^p < 0.0001 TEER values at 24 h vs. TEER values at 48 h, Two-way ANOVA, Bonferroni’s multiple comparison test). **D** Immunofluorescence images of hiPSC-derived BMECs. The image shows the maximum intensity projection of a Z stack. Blue: DAPI (nuclei), Green: ZO-1 (tight junctions). Scale bar 50 µm

**Fig. 3:**
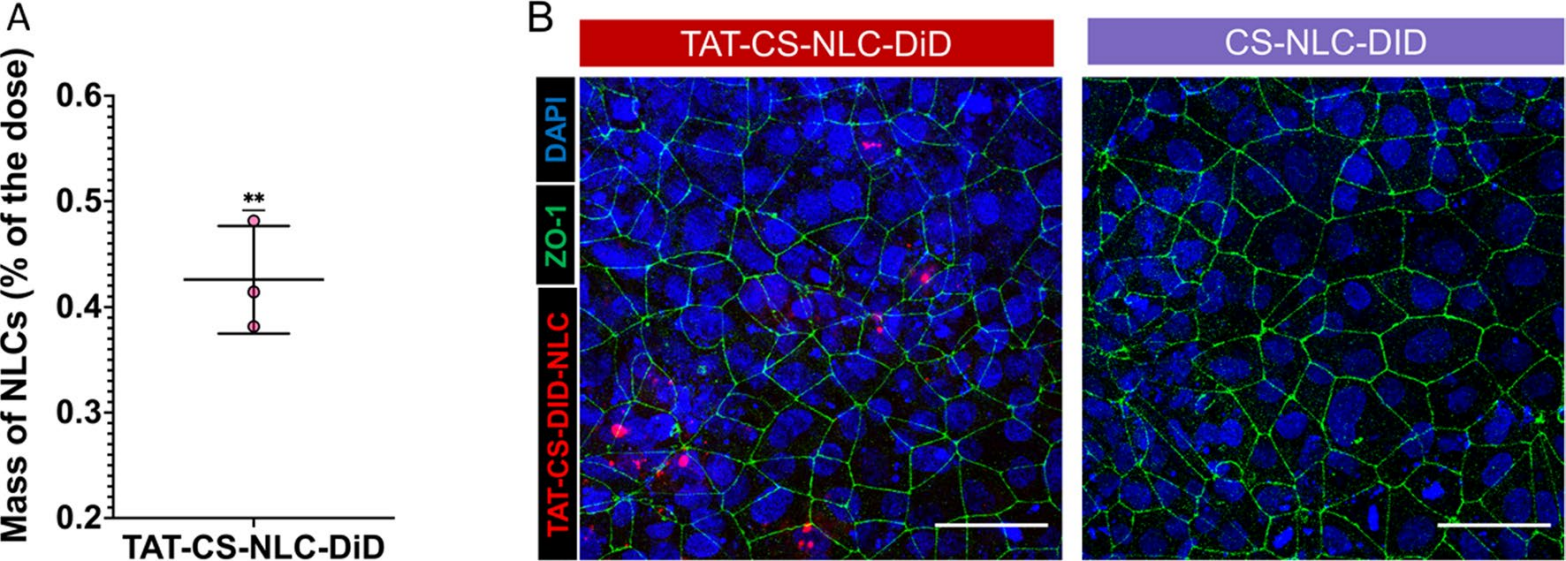
NLC modified transport across BMCEs **A** TAT-CS-NLC-DiD successfully penetrate the BMEC monolayer as opposed to the CS-NLC-DiD. (Data are means ± SEM of three independent experiments, p < 0.01 ^**^, One-sample t-test analysis) **B** Representative images of the BMEC monolayer after treatment with our NPs. Blue shows the nuclei stained with DAPI, green shows the tight junctions stained with ZO-1, whereas the NLCs are shown in magenta (incorporated DiD). TAT-CS-NLC-DiD could be detected in our cell monolayer, while CS-NLC-DiD were not detectable. The images show the maximum intensity projection of a Z stack. Scale bar 50 µm

To evaluate if surface modification with TAT peptide would enhance the NLC transport across the BBB, we performed a set of transport experiments. We exposed the hiPSC-derived BBB to the various NLCs for two hours and evaluated the NLC efficacy to cross the BMEC layer. Notably, our previous experiments confirmed barrier integrity during NLC exposure (Additional file 1: File 5). Briefly, TEER remained high (between 4000 and 5000 Ω*cm^2^ prior and post-exposure) throughout our assay while *P*_app_ for Cascade Blue was < 2*10^–7^ cm/s for all tested conditions (CB; CB + TAT-CS-DiD; CB + CS-DiD) (Additional file 1: File 5). For the transport experiments described here, hiPSCs were differentiated to BMECs following the protocol described in Fig. 2A (protocol described by Neal et al. 2019 [47] with slight modifications). Our hiPSC-derived BMECs showed the expected barrier phenotype; as shown in Fig. 2B, TEER values were around 2500 Ω*cm^2^ at 48 h post subculture for all three experiments, and cells expressed the BBB-specific tight junction protein ZO-1 (Fig. 2C, D). The exact TEER values of the hiPSC-derived BMECs employed in the permeability assay were the following: 2166.213 ± 23.783, 3154.470 ± 55.167 and 2822.400 ± 20.844 Ω x cm^2^ (Mean ± SEM). After barrier establishment, we tested the permeability of the NLCs across the BMEC monolayer. To do this, we compared the transport rate of TAT-CS-NLC-DiD (TAT modified NPs) versus CS-NLC-DiD (non-modified NPs). Cells were treated with 1 mg/ml NPs; a concentration we used in previous transport studies in nasal epithelium cell monolayer [55], without any observed toxic effect or alteration in BBB integrity post-incubation (Additional file 1: Files 1 and 5). Two hours post-NLC exposure, we detected 0.426% ± 0.051 of TAT-CS-NLC-DiD in the basolateral chamber of the transwells, whereas CS-NLC-DiD were not detectable (Fig. 3A). Hence, we can conclude that TAT-CS-NLC-DiD successfully crossed the barrier. Confocal microscopy further confirmed these results, where only TAT-CS-NLC-DiD could be detected in the BMEC monolayer (Fig. 3B). Overall, our data demonstrate that our NPs crossed the hiPSC-derived BBB after surface modification with TAT peptide.

### Microglia viability after incubation with NLCs

**Fig. 4:**
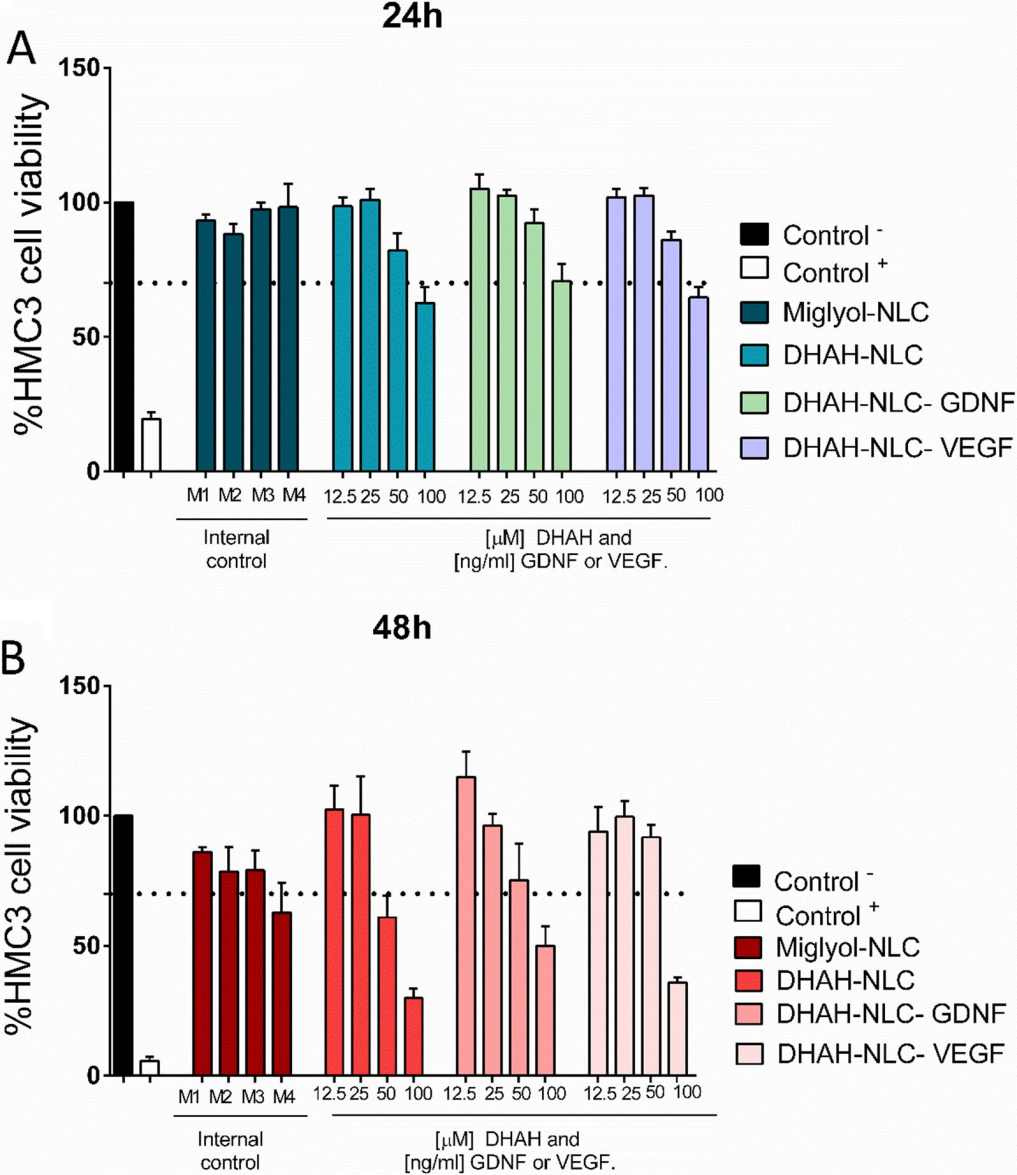
HMC3 cell viability study after incubation with the various NLCs (AlamarBlue reduction assay). **A** Cell viability after 24 h incubation with the various types and concentrations of NLCs (Additional file 1: File 3). **B** Cell viability after 48 h incubation with the various types and concentrations of NLCs (Additional file 1: File 3). In A and B, Control ^−^ denotes no treatment, media change and Control ^+^ denotes DMSO 10% for 24 h. Cell viability for each condition (treated cells) is expressed as the percentage of living cells compared to the nontreated cells (media change; Control ^–^), which was set as 100%. (Data are means ± SEM of three individual experiments; the dashed line represents 70% viability.)

To assess the cytocompatibility and the working concentration for our NPs, we performed an AlamarBlue viability assay after 24 h and 48 h incubation with the various NLCs. As shown in Fig. 4A, the incubation with the different NLCs led up to 70% cell viability at 24 h for all the tested conditions, except for when the highest concentration was used. We assume that the observed toxicity at 100 µM results from the high concentrations of DHA in both types of NLCs; DHA can be very toxic for the cells at the high concentration range (100–200 µM) [60, 61]. Interestingly, 48 h incubation of neuronal cultures with 100 µM DHA results in cell death, while lower concentrations lead to increased neuronal survival [60]. In our microglial cultures, we observed similar effects; as shown in Fig. 4B, after 48 h of incubation, only low concentrations (25 and 12.5 μM, for DHAH lipid and 25 and 12.5 ng/ml for GF, see also Additional file 1: File 3) led to high cell viability (> 70%). Thus, as working concentrations for the following experiments performed with the HMC3 human microglial cell line, we set 25 µM for the functional lipid DHAH and 25 ng/ml for the GFs for all the different NLCs containing DHAH functional lipid and GDNF or VEGF. In the case of Miglyol-NLCs, an equal dose of NLC was used, equivalent to 31 µg/ml for NLC concentration (M2; see also Additional file 1: File 3).

### DHAH-NLC modulate the microglial inflammatory response

#### Gene expression analysis (RT-qPCR)

We performed gene expression analysis in two different conditions to investigate if the NPs could modulate the inflammatory responses. First, in preconditioning assay, we tested if the interaction between the microglia and the NPs would induce any inflammatory responses in the microglial cultures. Here, cells were treated with the various NLCs (Miglyol-NLC, DHAH-NLC, DHAH-NLC-GDNF, DHAH-NLC-VEGF) or LPS (Control + ; control for inflammation induction) for 24 h and samples were collected for gene expression analysis. As a negative control (Control –), we used cells with only media change (Additional file 1: File 6). Next, we tested if the NPs could modulate inflammation after LPS *stimuli* in the anti-inflammatory assay. In this case, the NLC pre-treated groups (Miglyol-NLC, DHAH-NLC, DHAH-NLC-GDNF, DHAH-NLC-VEGF) were challenged with LPS for 24 h. Similar to preconditioning assay, as a control for the inflammatory state, we used cells that received only LPS (Control +) and as negative control just media change (Control ^–^) (see also Additional file 1: File 6).

As shown in the bright-field images in Additional file 1: File 6, LPS stimulation resulted in a shift of the microglia towards an ameboid phenotype (Control^+^; Additional file 1: File 6). Notably, this effect was not observable in the other groups, which we treated with the various NLCs. Our analysis included both proinflammatory genes (IL-6, TNFα, IL1-β, NF-κB, COX-2) and antioxidant genes (Nrf2, HO-1).

##### Preconditioning assay

**Fig. 5:**
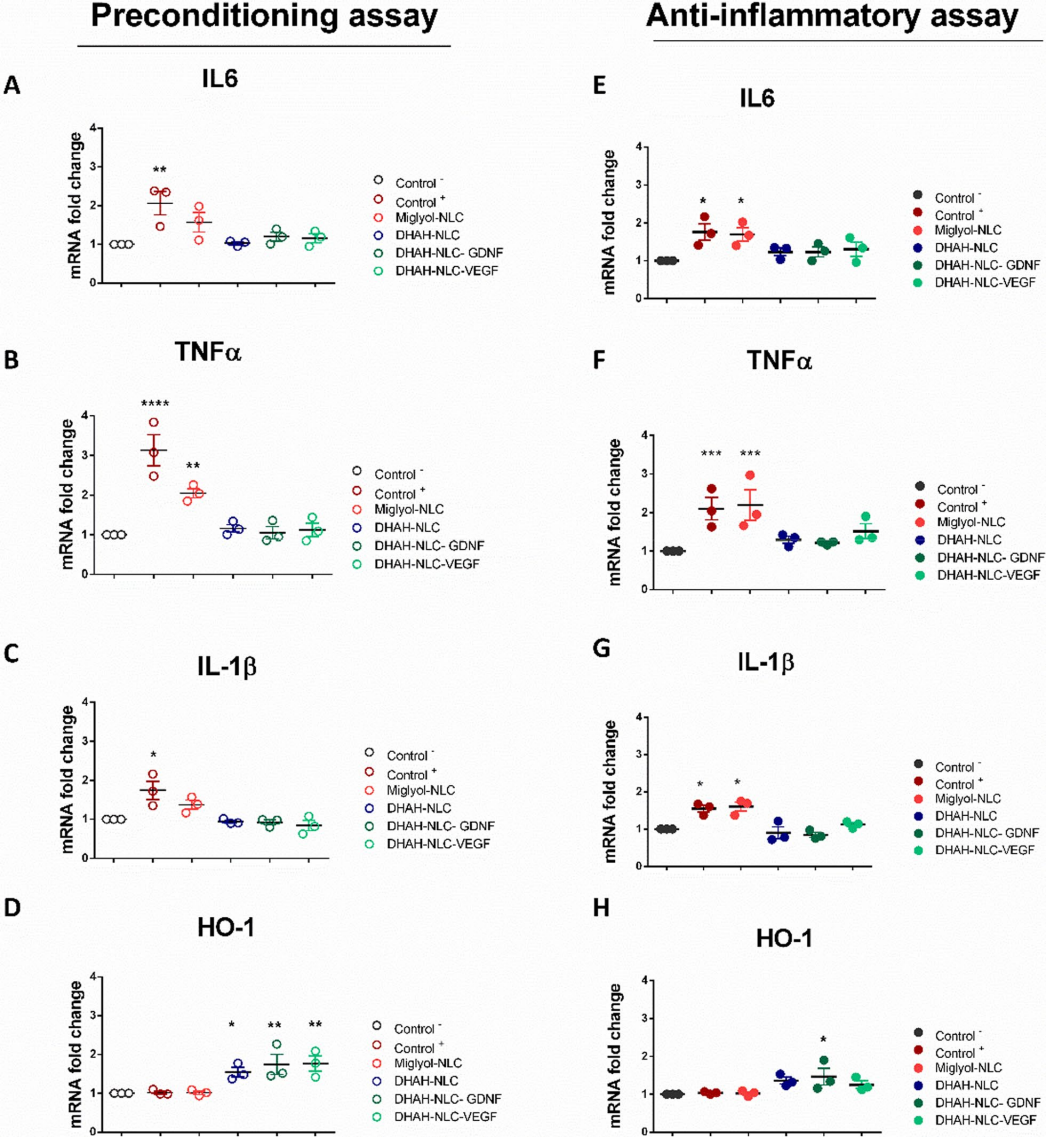
Gene expression analysis (RT-qPCR). **A** IL6 **B** TNF-α **C** IL-1β and **D** HO-1 for preconditioning assay. **E** IL6 **F** TNF-α **G** IL-1β and **H** HO-1 for anti-inflammatory assay (see also Additional file 1: File 9). Relative mRNA expression was normalized against GAPDH, and the gene expression of the group where only media change was performed (Control^−^) was used as a reference (fold change 1). (Data are means ± SEM of three individual experiments; ΔCT values were used for statistical analysis, ^*^p < 0.05 ^**^ p < 0.01 ^***^ p < 0.001. One-Way ANOVA, Bonferroni’s multiple comparison test; each group mean *vs.* the mean of Control ^−^. In both conditions, Control ^−^ denotes no treatment, media change and Control ^+^ denotes LPS incubation (100 ng/ml)

*Proinflammatory response*: LPS treatment for 24 h upregulated IL-6, TNF-α, and IL-1β (Fig. 5), while the levels of COX-2 and NF-κβ remained unchanged (Additional file 1: File 8). The LPS treated cells showed higher mRNA expression of IL-6 levels than the untreated, while treatment with the various NLCs did not significantly change. The Miglyol-NLC treated group showed a slight trend towards increase, but the effect did not reach significance. The DHAH-NLC, DHAH-NLC-GDNF, and DHAH-NLC-VEGF treated cells exhibited similar expression levels to the untreated cells with values significantly lower than the LPS treated cells (Fig. 5A). For TNF-α, we observed a similar response with IL-6; LPS treatment resulted in the upregulation, whereas incubation with DHAH-NLC, DHAH-NLC-GDNF, and DHAH-NLC-VEGF did not induce any changes. Here, in the Miglyol-NLC treated group, we observed a significant increase compared to the untreated. The DHAH-NLC, DHAH-NLC-GDNF, and DHAH-NLC-VEGF treated cells exhibited similar expression levels to the untreated cells; values were significantly lower than the LPS and the Miglyol-NLC treated cells (Fig. 5B). Finally, IL-1β mRNA levels increased following LPS stimulation, while none of the various NLCs, affected gene expression. The DHAH-NLC, DHAH-NLC-GDNF, and DHAH-NLC-VEGF treated cells exhibited similar expression levels to the untreated cells with values significantly lower than the LPS treated cells (Fig. 5C).

*Antioxidative response*: Here, we investigated if our NPs could induce any antioxidative responses in our microglial cultures. Thus, we tested for alterations in the gene expression profile of the cultures for two traditional antioxidant genes, Nrf2 and HO-1. Administration of all the DHAH-enriched NLCs increased the levels of HO-1, but Nrf2 expression did not change. Furthermore, both Nrf2 and HO-1 mRNA expression levels remained unchanged after treatment with Miglyol-NLC (Fig. 5D, Additional file 1: Files 8C and 9D).

##### Anti-inflammatory assay

*Proinflammatory response*: As expected, LPS *stimuli* resulted in IL-6 upregulation, which was not reverted after treatment with Miglyol-NLC. In contrast, incubation with DHAH-NLC, DHAH-NLC-GDNF, and DHAH-NLC-VEGF led to significant downregulation (Fig. 5E). Furthermore, we observed a similar scheme for TNF-α; LPS increased mRNA expression levels, while following treatment with Miglyol-NLC did not revert the effect. On the contrary, treatment with DHAH-NLC and DHAH-NLC-GNDF led again to downregulation, with TNF-α levels being significantly lower than both the LPS treated and the Miglyol-NLC group. We observed a similar trend for the DHAH-NLC-VEGF treated cells, but this effect did not reach significance (Fig. 5F, Additional file 1: File 9F). Lastly, following a similar pattern to the previous results, LPS treatment induced IL-1β upregulation, which was not counteracted after treatment with Miglyol-NLC. However, treatment with DHAH-NLC and DHAH-NLC-GDNF reverted this effect; the levels were lower than the LPS treated cells and similar to the untreated cells. Moreover, in the case of IL-1β, we observed a slight decrease in the DHAH-NLC-VEGF treated cells, but the effect was not significant (Fig. 5G, Additional file 1: File 9G).

*Antioxidative response:* Here, only DHAH-NLC-GDNF incubation led to observable changes in HO-1 expression. For the other DHAH-enriched NLCs, although we observed a slight trend for an increase, it did not reach significance. Miglyol-NLC treated cells showed no change in HO-1 levels (Fig. 5H). Nrf2 levels remained unchanged in all the tested groups (Additional file 1: File 8F).

#### Multiplex assay

From the U-plex assay, only IL1-β, IL-6 and IL-8 could be detected in cell culture supernatant in all conditions.

##### Preconditioning assay

**Fig. 6:**
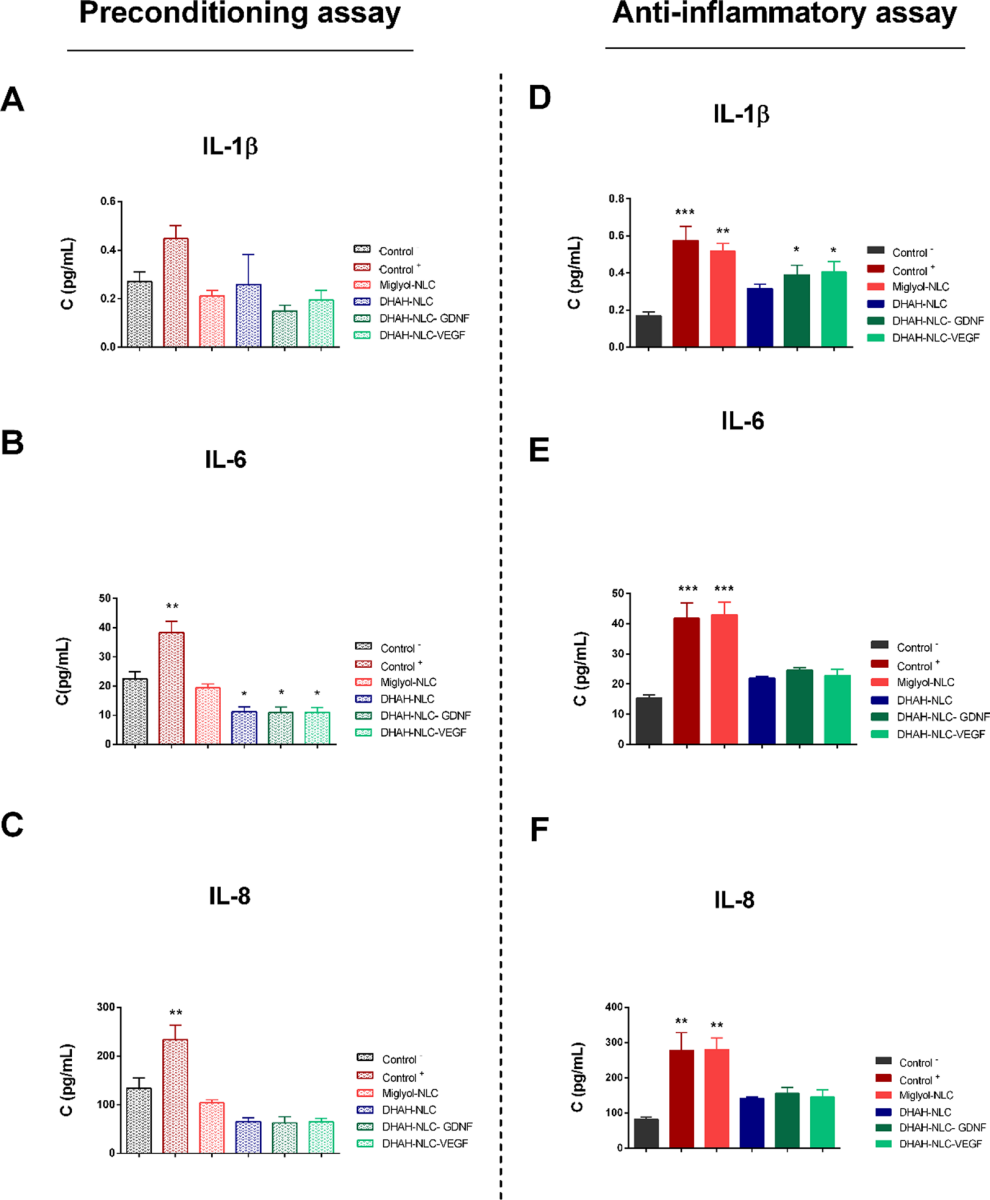
Cytokine secretion analysis (U-PLEX Assay) assay values. **A** IL-1β **B** IL-6 and **C** IL-8 for preconditioning assay. **D** IL-1β **E** IL-6 and **F** IL-8 for anti-inflammatory assay (see also Additional file 1: File 10). (Data are means ± SEM of three individual experiments. ^*^p < 0.05 ^**^p < 0.01 ^***^p < 0.001, ^****^p < 0.0001, One-Way ANOVA, Bonferroni’s multiple comparison test; each group mean *vs* the mean of Control ^−^). In both conditions, Control ^−^ denotes no treatment, media change and Control ^+^ denotes LPS incubation (100 ng/ml)

In this condition, treatment with LPS for 24 h led to an increase in cytokine secretion of IL-6 and IL-8 (Control + ; Fig. 6A–C). IL-1β showed a similar trend, but it did not reach significance. Incubation with Miglyol-NLC did not alter cytokine secretion (Fig. 6A–C) compared to the basal levels (Control-). Cells treated with the DHAH formulation (with or w/o GFs) secreted the same level of IL-1β (Fig. 6A). Interestingly, secretion of IL-6 was significantly lower than the control (Fig. 6B), highlighting the potential anti-inflammatory properties of the DHAH enriched formulations (with or w/o GFs).

##### Anti-inflammatory assay

Cells pre-treated with the DHAH-enriched nanoformulations counteracted LPS-induced secretion of cytokines. All detected cytokines exhibited significantly reduced secretion (Fig. 6D–F), showing similar levels to Control ^–^ compared with this group. Miglyol-NLC pre-treated cells showed no significant difference upon stimulation compared to LPS-only treated cells. Overall, these results demonstrate the immunomodulatory effect that the DHAH formulation elicits. Encapsulation of GFs did not seem to have a pronounced impact on the inflammatory profile upon LPS stimulation (Additional file 1: File 10). In detail, the secretion of all detected cytokines was on par with the DHAH-NLC treated group (Fig. 6D–F).

## Discussion

The lack of effective treatments for NDs raises an urgent need to identify new drug candidates. During the last years, different agents ranging from GFs to more natural compounds such as PUFAs have been suggested as feasible options to manage neurodegenerative processes [17, 62]. Nonetheless, brain targeting remains challenging due to the BBB, a tight barrier of the BMECs regulating flux in and out of the brain [63]. To achieve the delivery of neurotherapeutics into the brain, scientists have followed various strategies during the last decade [64, 65]. To this end, in the present study, we employed human-oriented in vitro models to investigate if TAT-functionalized DHAH-based NLCs could (a) surpass the human BBB and (b) modulate neuroinflammation.

The developed and characterized NLCs used in this work showed similar characteristics to those reported in our previous studies regarding their size, uniform appearance, and encapsulation efficiency [27, 28, 33, 55]. Transport via the BBB is challenging; moreover, the poor barrier properties of most BBB models render transport studies virtually impossible. Among the different models to mimic the human BBB, hiPSC-derived BBB-like cells form tight barriers with high TEER and low paracellular diffusion; thus, they offer promising in vitro platforms for drug transport and screening assays [37, 38, 47, 58, 66–68]. HiPSC-derived BMECs mimic the human brain microvasculature to a large extent; hence, they are an ideal system to investigate if the NLCs can efficiently bypass the human BBB and reach the CNS. Using this physiologically relevant, human in vitro model, we reported that TAT-modified nanoparticles demonstrated enhanced transport capability with ~ 0.43% of the dose of the particles crossing the BBB-like layer (Fig. 4). This data highlights the potential of the TAT-modified NLCs to target the human brain. The percentage of the transported particles is low; yet, to our knowledge, it is the highest reported using a human, physiologically relevant in vitro model. To date, most permeability studies for NLCs have utilized the bEnd.3 cell line as a BBB in vitro model. Other studies have shown higher transport rates for similar agents across the BBB than the one we reported here, but the barrier properties of their models were suboptimal. In those studies, TEER values range from 150 to 400 Ω × cm^2^, while the minimum for in vitro drug screening models, according to Mantle et al., is 900 Ω × cm^2^ [69]. For example, Dos Santos Rodriguez et al. reported 2–3% transport of TAT-modified liposomes two hours post-exposure using a BBB in vitro model with a TEER value of 400Ω × cm^2^ [70]. In bEnd.3 cell monolayers, Jiang et al. made similar observations for polymeric nanoparticles (NP) modified with the facilitative glucose transporter (GLUT) (NP-GLUT) [71]. In the case of α2-macroglobulin-modified polymeric NP, Cox et al. report that 15% of their nanoformulation crossed the BBB in vitro model with a TEER value of 900 Ω × cm^2^ [72]. Although these transport rates are higher than the ones shown in this study, these studies lack a vital element: barrier tightness. Therefore, this data may show lower translatability and clinical significance. Our results, on the contrary, originate from studies performed in an in vitro BBB model with high TEER values ranging from 2000–5000 Ω × cm^2^, low paracellular diffusion, and the expression of tight junction proteins (Fig. 2 and Additional file 1: Files 5, 11 (comparison to primary brain microvascular cells)). These characteristics mimic to a large extent the barrier properties in vivo; thus, our results may show higher translatability to the clinic. To our knowledge, there is only one published work where the authors employed a hiPSC-based BBB model in transport studies; however, they were not able to distinguish between atorvastatin in solution and PLGA-based atorvastatin-loaded NPs [73]. Indeed, previous studies conducted by our research group showed that polymeric nanoparticles, such as PLGA NPs, are not the best nanotechnology approach to cross-physiological barriers. Gartziandia et al. reported that lipid nanoparticles, named NLCs, had better results crossing the nasal epithelium barrier. Moreover, the surface modification with TAT peptide enhances its ability to cross this physiological barrier [55]. The exact mechanism that regulates cellular uptake of TAT peptide remains unclear; studies support show that it is a result of various mechanisms including endocytosis, transcytosis, micropinocytosis and could be affected by many factors such as, among others, cargos, and specific stimuli [74–76].

In summary, the data presented here show that lipid nanoparticles and their surface modification with TAT peptide offer a nanotechnology-inspired approach to enhance transport across physiological barriers and, specifically, an in vivo-like in vitro BBB model. Thus, this study brings us a step forward in attaining brain targeting. It is arguable if the observed transport rate of 0.5% is clinically relevant. As we stated above, the quantity of particles that reached the brain compartment in our model is low; however, it is higher than those observed in previous in vivo studies with similar nanoformulations. For example, Beloqui et al. reported that only 0.01% of the nanoformulation accumulated in the brain after intravenous injection in rats [77].

Moreover, previous in vivo studies carried out by Hernando et al. with TAT peptide- and CS-modified nanoparticles demonstrated their ability to target and treat MPTP-lesioned mice. NLC treatment increased GDNF levels, restored motor activity, increased TH expression, and modulated the microgliosis present in a model of Parkinson’s disease. Therefore, we hypothesize that nanoparticles that reach the brain could exert their therapeutic function [27].

To test the potential anti-inflammatory and antioxidative effect of these nanoparticles (w or w/o GF) once they reach the brain, we tested their ability to regulate microglial responses in a human-relevant context. Thus, we performed the current study using HMC3, a human microglial cell line as in vitro system, and LPS as an inflammation inducer [78]. LPS is used widely in both in vivo and in vitro models to induce inflammation in microglia [79–81], while others have previously shown that HMC3 also responds to LPS [82, 83]. We, therefore, concluded that HMC3 was a suitable experimental model for this work. Indeed, our data show that HMC3 were responsive to LPS stimuli manifested by an altered gene expression profile and cytokine response compared to basal conditions (Figs. 5, 6).

Upon LPS treatment, microglia shifted to a pro-inflammatory state showing IL-6 response, in agreement with previous reports [78]. Interestingly, in our study, IL-8 cytokine exhibited the highest values with a three-fold upregulation after the incubation with LPS; this effect has been observed previously after the incubation with NS3 protein but not after LPS incubation [84]. Despite the observed upregulation of TNF-α at the gene expression level, we could detect cytokine release in the media with the U-PLEX assay. Moreover, the qPCR analysis did not show any effects in COX2 and NF-κβ expression (Additional file 1: File 8), which is in line with previous publications after different inflammatory stimuli [84, 85]. Taken together, our data show that the HMC3 microglial cell line responds to LPS stimuli; therefore, we utilized these cells as an in vitro platform to test our NLCs for their ability to modulate inflammatory processes and provide data with human relevance.

Next, we investigated if treatment with various NLCs could prevent the LPS-induced inflammatory responses. Indeed, as shown in Fig. 5E–G and Fig. 6D–F, 24 h incubation with DHAH-NLCs counteracted inflammatory response in the microglia; an effect that we did not observe after incubation with Miglyol-NLC. Our results highlight the potential DHAH-NLC treatment halting the undergoing neuroinflammation and point out that the anti-inflammatory properties of Ω-3 fatty acids are maintained when formulated in the NLC lipid matrix [86, 87]. Furthermore, previous studies suggest that GDNF and VEGF can inhibit neuroinflammation and, therefore, protect from neurodegeneration [88, 89]. Hence, we hypothesized that the encapsulation of these GFs in our DHAH-NLCs would further promote their anti-inflammatory potential. Surprisingly, the encapsulation of GDNF or VEGF in the DHAH enriched NLCs (DHAH-NLC-GDNF, DHAH-NLC-VEGF) did not seem to have a pronounced effect on the inflammatory profile upon LPS stimulation (Figs. 5, 6). In detail, the secretion of all detected cytokines was on par with the DHAH-NLC condition suggesting that either this specific microglia source is not responsive to GFs or that these GFs manifest their anti-inflammatory properties in other pathways [90, 91]. While, to our knowledge, the effect of VEGF in activated microglia remains elusive [92], a recent study suggests that GDNF regulates microglia responses via the activation of the endogenous anti-oxidative system and not due to the downregulation of pro-inflammatory markers such as TNF-α or IL-6 [93].

The antioxidant pathway Nrf2/HO-1 is a putative target against oxidative stress and neuroinflammation in NDs [94, 95]. We, therefore, investigated if our NPs could show antioxidant abilities via this molecular cascade. Our data showed that treatment with all the DHAH enriched NLCs upregulated HO-1 in basal conditions (Preconditioning assay, Fig. 5D, Additional file 1: File 9). Additionally, DHAH-NLC-GDNF was the only condition that showed a significant upregulation in HO-1 levels upon LPS *stimuli,* while the rest of the DHAH enriched nanoformulations induced a slight increase that did not reach significance. Thus, we concluded that DHAH-NLC-GDNF activated the anti-oxidative system present in microglia, which is in line with recent studies [93, 96].

## Conclusions

Taken together, using a hiPSC-derived BBB model featuring high TEER values and low paracellular transport, we show here that TAT-functionalized DHAH-NLCs can cross the BBB and act as trojan horses for potential therapeutics to the CNS. Moreover, DHAH-NLCs counteracted LPS-induced inflammation and oxidative stress in human microglial cultures, while GDNF encapsulation in the particles further enhanced the anti-oxidative properties of the microglia. Hence, we suggest TAT-functionalized DHAH-NLCs as novel drug delivery systems that effectively cross the BBB and potentially treat human CNS disorders.

## Supplementary Information

**Additional file 1: File 1.** Methods. **File 2.** Summary of the reagent used in this research article. **File 3.** Composition of the different type of NLCs and doses tested in HMC3 microglia cell line. **File 4.** Taqman assay ID. **File 5.** Barrier integrity post-NLC exposure. **File 6.** NLC incubation-working concentrations set in AlamarBlue assay- in HMC3 microglia cell line. **File 7.** HMC3 cell viability after exposure to LPS or NLC (Miglyol-NLC, DHAH89 NLC, DHAH-NLC-GDNF, DHAH-NLC-VEGF in working concentrations). **File 8.** Gene expression analysis (RT-qPCR). **File 9.** Gene expression analysis (RT-qPCR). **File 10.** Cytokine secretion analysis (U-PLEX Assay) assay values. **File 11.** NLC permeability across the human BBB.

## Data Availability

Data analyzed in the current study are available from the authors on reasonable request.

## Abbreviations

AD: Alzheimer’s disease
ANOVA: Analysis of variance
BBB: Blood–brain barrier
BMECs: Brain microvascular endothelial cells
CNS: Central nervous system
COX-2: Ciclooxigenase-2
CPP: Cell penetrating peptide
CS: Chitosan
DAPI: 4′,6-Diamidino-2-phenylindole
DDS: Drug delivery system
DiD: 1,1'-Ioctadecyl-3,3,3',3'-Tetramethylindodicarbocyanine Perchlorate
DHA: Docohexaenoic acid
DHAH: Hydroxilated derivate of docohexaenoic acid
DLS: Dynamic light scattering
DMEM: Dulbecco's modified eagle medium
DMSO: Dimethyl sulfoxide
DPBS: Dulbecco's phosphate-buffered saline
EDC: 1-Ethyl-3-(3-dimethylaminopropyl) carbodiimide hydrochloride
EE: Encapsulation efficiency
ELISA: Enzyme-Linked ImmunoSorbent Assay
GAPDH: Glyceraldehyde-3-phosphate dehydrogenase
GDNF: Glial derived neurotrophic factor
GF: Growth factor
GFP: Green fluorescent protein
GFAP: Glial fibrillary acidic protein
HBSS: Hank's balanced salt solution
hESFM: Human endothelial serum free medium
hiPSCs: Human induced pluripotential stem cells
HO-1: Hemoxygenase
IL-6: Interleukin-6
IL-8: Interleukin-8
IL-1β: Interleukin- 1β
LPS: Lipopolysaccharide
ND: Neurodegenerative disease
NF-κB: Nuclear factor B
NLC: Nanostructured lipid carrier
NP: Nanoparticle
Nrf2: Nuclear factor erythroid 2–related factor 2
NTF: Neurotrophic factor
NVU: Neurovascular unit
ON: Overnight
PBS: Phosphate buffer saline
PCR: Polymeric chain reaction
PD: Parkinson’s disease
PDI: Polydispersity index
PEG: Polyethylene glycol
PFA: Paraformaldehyde
P/S: Penicillin/streptomycin
PUFAs: Polyunsaturated fatty acids
RA: Retinoic acid
ROS: Reactive oxygen species
RNA: Ribonucleic acid
RT: Room temperature
SEM: Standard error of mean
sulfo-NHS: N-Hydroxysulfosuccinimide
TAT: Transactivator of transcription
TEER: Transendothelial electrical resistance
TEM: Transmission electron microscopy
TNF: Tumor necrosis factor α
VEGF: Vascular endothelial growth factor
WHO: World Health Organization
ZO-1: Zonula occludens-1
