# Supplementary material for: Dual effect of TAT functionalized DHAH lipid nanoparticles with neurotrophic factors in human BBB and microglia cultures
Source: Fluids Barriers CNS. 2022 Mar 17;19:22. doi: 10.1186/s12987-022-00315-1 (PMC8928663; doi:10.1186/s12987-022-00315-1)
Supplement: Supplementary file 1 — Additional file 1: File 1. Methods. File 2. Summary of the reagent used in this research article. File 3. Composition of the different type of NLCs and doses tested in HMC3 microglia cell line. File 4. Taqman assay ID. File 5. Barrier integrity post-NLC exposure. File 6. NLC incubation-working concentrations set in AlamarBlue assay- in HMC3 microglia cell line. File 7. HMC3 cell viability after exposure to LPS or NLC (Miglyol-NLC, DHAH89 NLC, DHAH-NLC-GDNF, DHAH-NLC-VEGF in working concentrations). File 8. Gene expression analysis (RT-qPCR). File 9. Gene expression analysis (RT-qPCR). File 10. Cytokine secretion analysis (U-PLEX Assay) assay values. File 11. NLC permeability across the human BBB. [file 12987_2022_315_MOESM1_ESM.pdf]

Additional Information

Dual effect of TAT functionalized DHAH lipid nanoparticles with neurotrophic factors in human BBB and microglia cultures

Sara Hernando+ [a, b, e, f, g, 1] , Polyxeni Nikolakopoulou+ [a, b], Dimitrios Voulgaris [a, b, c, d], Rosa Maria Hernandez [e, f, g], Manoli Igartua [e, f, g, *], Anna Herland [a, b, c *]

[a] Center for the Advancement of Integrated Medical and Engineering Sciences (AIMES) Karolinska Institutet and KTH Royal Institute of Technology Stockholm SE-171 77 Sweden.

[b] Department of Neuroscience Karolinska Institutet Stockholm SE-171 77 Sweden.

[c] KTH Royal Institute of Technology, Division of Nanobiotechnology, Department of Protein Science, Science for Life Laboratory, Solna, Sweden.

[d] Division of Micro and Nanosystems, KTH Royal Institute of Technology, Stockholm, Sweden

[e] NanoBioCel Research Group, Laboratory of Pharmaceutics, School of Pharmacy University of the Basque Country (UPV/EHU), 01006 Vitoria-Gasteiz, Spain

[f] Biomedical Research Networking Centre in Bioengineering, Biomaterials and Nanomedicine (CIBER-BBN), Institute of Health Carlos III, 28029, Madrid Spain

[g] Bioaraba, NanoBioCel Research Group, 01006, Vitoria-Gasteiz, Spain

(+) Shared authors

(*) Co-corresponding author

Manoli Igartua: (+34) 945 01 3007 manoli.igartua@ehu.eus ; Anna Herland (+46) 8 790 84 31 aherland@kth.se

---

[1] Permanent address:

[e] NanoBioCel Research Group, Laboratory of Pharmaceutics, School of Pharmacy University of the Basque Country (UPV/EHU), 01006 Vitoria-Gasteiz, Spain

[f] Biomedical Research Networking Centre in Bioengineering, Biomaterials and Nanomedicine (CIBER-BBN), Institute of Health Carlos III, 28029, Madrid Spain

[g] Bioaraba, NanoBioCel Research Group, 01006, Vitoria-Gasteiz, Spain

Additional File 1

Methods

Barrier integrity after NLC exposure (additional file 5)

To assess barrier integrity during NLC treatment, we employed tracer dye permeability, ICC and confocal imaging. hiPSC-derived BMECs were seeded on Transwell filters ($3.3x10^5$ cells per filter), and 48h post subculture TEER was measured to evaluate barrier formation. Next, media was replaced with fresh, pre-warmed media and cells were incubated at 37°C for 2 hours to compensate for volume loss due to evaporation and ensure temperature stabilization. Post the two-hour incubation, BMECs were transferred on an orbital shaker (37°C), and permeability assay was performed. During the permeability assay, cells were exposed to CB (100μgr/ml) ± the different NLCs (1mgr/ml). CB is an optimal paracellular marker due to its small size and high hydrophilicity (MW 596.44, log $P \approx -7$) [1] .

The apparent permeability (P_{app}) of CB across the BMEC monolayer was assessed after 4h exposure for the three different conditions (CB only, CB+TAT-CS-DiD mix and CB+CS-DiD). Briefly, at 2h, 3h and 4h of exposure 50μl media was collected from the basolateral chamber (after pipetting to ensure that samples were properly mixed) and placed to a 96-well plate. Missing volume was replaced with 50μl fresh medium at the bottom chamber. At the last time point, i.e., 4h exposure, 50μl medium was collected from the apical chamber, diluted 1:3 in media and transferred to the 96-well plate. CB fluorescence was read on a plate reader (Plate Reader Infinite M1000, Tecan, Switzerland; 400nm excitation/420 emission). Apparent permeability (P_{app}) of CB was calculated according to the formula

$$Papp = \frac{dS_b}{dt} * \frac{V_b}{A * S_a}$$

for both the culture and the blank filter (no cell layer, similar treatments) [2]. Experiments were performed in triplicates or quadruplicates (different Transwells from one differentiation) depending on the condition.

To further verify the integrity of the BMEC layer upon particle exposure, we performed TEER measurements prior and post permeability assay. At the end of the experiment, Transwell filters

were fixed and stained (following the procedure described in the methods section) to verify a) particle uptake and b) tight junction (ZO-1 expression) and cell membrane integrity (Phalloidin staining). BMEC layers were visualized using a laser scanning confocal microscope (Zeiss LSM800, 40X and 100X objective).

NLC permeability across the human BBB (hiPSC-BMVECs vs human primary BMEC, additional file 11)

To access NLC barrier permeability across the different BBB cell models we performed permeability assay for 2h in both primary human endothelial brain microvascular cells (phBMECs; ACBRI 376, Cell Systems) and hiPSC-derived BMECs (ihBMECs). phBMECs were seeded on attachment factor coated Transwell filters (150000 cells/cm^2, passage 8 in Endothelial Cell Growth Medium MV2) while ihBMECs were seeded as described above. 48 h post seeding TEER was assessed for both cell types, and permeability assay was performed as described above with minor modifications (2h timepoint, sampling volume 200ul basolateral/10ul apical, apical further diluted 1:100). TEER values for the cells used in this experiment were 2844 ± 468 Ω x cm^2 for the ihBMECs and 13.96 ± 8.16 Ω x cm^2 for the phBMECs. Tracer dye (CB) permeability was utilized to evaluate barrier integrity for the two different BBB models during NLC exposure. CB and NLC P*app* were calculated after fluorescence measurement on a plate reader (Plate Reader Infinite M1000, Tecan, Switzerland; excitation/emission 400/420 for CB and 644/655 for DiD). At the end of the experiments, Transwells were fixed and stained with Hoechst and Phalloidin to evaluate particle uptake. BMECs were visualized using a laser scanning confocal microscope (Zeiss LSM880, 63X objective).

Additional file 2 Summary of the reagent used in this research article

PRODUCT	Reference (company)
**Precirol ATO®5** (glycerol distearate)	Gattefosé
**Miglyol ®** (caprylic/capric triglyceride)	Sasol Germany GmbH
**DHAH fatty acid** (ethyl ester form)	Medalchemy
**Tween 80**	162050 (Panreac)
**Tween 20**	62312 (Panreac)
**Lutrol ® F-68 (Poloxamer 188)**	9003-11-6 (VWR)
**Trehalose dehydrate**	T5251 (Sigma)
**Chitosan (Ultrapure)**	4210021 (Novamatrix)
**Matrigel**	354230 (Corning)
**mTeSR ™ 1 media**	85850 (Stem cell technologies)
**ROCK inhibitor**	1254 (TOCRIS)
**Tryple Select**	12563011 (Gibco)
**Versene**	15040066(Gibco)
**E6 media (Essential 6 medium)**	A1516401 (Gibco)
**hESFM media (human endothelial serum free media)**	11111044 (Gibco)
**B27 ™ supplement (50X)**	17504044 (Gibco)
**bFGF (basic fibroblastic growth factor)**	233-FB (R&D Systems)
**RA (retinoic acid)**	R2625 (Sigma)
**Fibronectin**	F1141 (Sigma)
**Collagen type IV**	C 5533 (Sigma)
**DMEM/F-12, GlutaMAX™**	31331028 (Gibco)
**Fetal bovine serum (FBS)**	A3840202 (Gibco)
**Dulbecco's phosphate-buffered saline (DPBS), calcium, magnesium**	14080048 (Gibco)
**Dulbecco's phosphate-buffered saline (DPBS) no calcium, no magnesium**	14190094 (Gibco)
**4′,6-diamidino-2-phenylindole (DAPI)**	D1306 (Invitrogen)

**1,1'-Dioctadecyl-3,3,3',3'-Tetramethylindodicarbocyanine Perchlorate**	D307 (Invitrogen)
**Paraformaldehyde (PFA)**	441244 (Sigma)
**Triton ™ x-100**	X100 (Sigma)
**Normal Goat serum (NGS)**	G9023 (Sigma)
**β-Mercaptoethanol**	31350-010 (Sigma)
**ZO-1 antibody**	33-9100 (Invitrogen)
**Anti-Mouse IgG1 (γ1), CF™488A**	SAB4600237 (Sigma)
**LPS-EK Ultrapure**	tlrl-peklps (InvivoGen)
**RNA isolation kit**	11 828 665 001 (Roche)
**High-Capacity RNA-to-cDNA™ Kit**	4387406 (Applied Biosystems)
**TaqMan ™ Fast Advanced Master Mix**	*4444557* (Applied Biosystems)
**VEGF (vascular endothelial growth factor)**	100-20 (Peprotech)
**GDNF (glial derived growth factor)**	450-10 (Peprotech)
**VEGF ELISA Kit**	900-K10 (Peprotech)
**GDNF ELISA Kit**	Ab100525 (Abcam)
**TAT peptide**	ChinaPeptides
**Cascade Blue™ hydrazide, Trisodium Salt**	C687 (Thermo Fisher)
**Alexa Fluor™ 488 Phalloidin**	A12379 (Thermo Fisher)
**Primary endothelial cells (ACBRI 376)**	Cell Systems
**Hoechst 33342, trihydrochloride, trihydrate**	H1399 (Thermo Fisher)
**Attachment factor**	S006100 (Thermo Fisher)
**Endothelial Cell Growth Medium MV2**	PromoCell

Additional file 3 Composition of the different type of NLCs and doses tested in HMC3 microglia cell line.

**Miglyol-NLC**				**DHAH-NLC**				**DHAH-NLC-GDNF**				**DHAH-NLC-VEGF**			
15.5	*31*	*62*	*124*	*12.5*	*25*	*50*	*100*	*12.5*	*25*	*50*	*100*	*12.5*	*25*	*50*	*100*

Internal control *(M1-M4)*(µg/ml)	+	+	+	+	-	-	-	-	-	-	-	-	-	-	-	-
DHAH (µM)	-	-	-	-	+	+	+	+	+	+	+	+	+	+	+	+
GDNF (ng/ml)	-	-	-	-	-	-	-	-	+	+	+	+	-	-	-	-
VEGF (ng/ml)	-	-	-	-	-	-	-	-	-	-	-	-	+	+	+	+

Additional file 4 Taqman assay ID

Gene name	***Taqman assay ID***
**GADPH**	Hs04420697_g1
**NrF2**	Hs00975961_g1
**HO-1**	Hs01110250_m1
**NF-κB**	Hs00765730_m1
**IL-1β**	Hs00174097_m1
**IL-6**	Hs00985639_m1
**TNF-α**	Hs00174128_m1
**COX-2**	Hs00153133_m1

Additional file 5 Barrier integrity

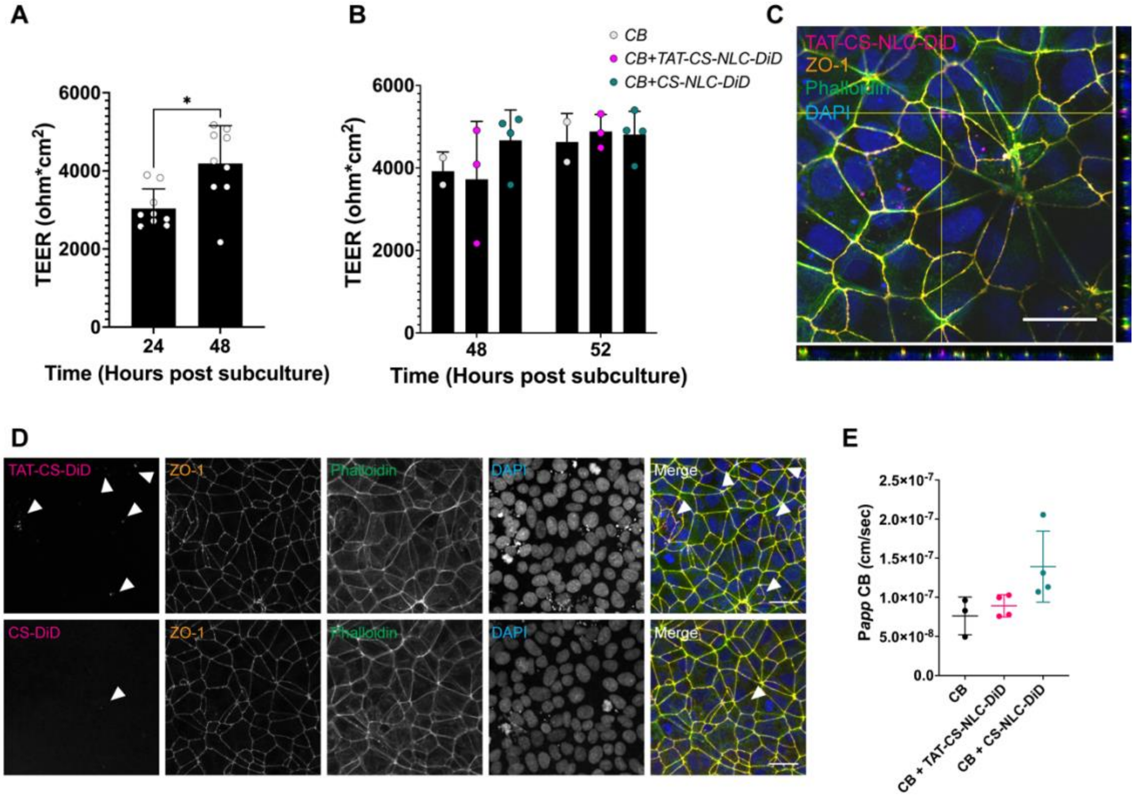

Additional file 5 Barrier integrity post-NLC exposure. (A) TEER values at 24h and 48h after subculture onto Transwells. (Data are means ± SD of nine different Transwells. *p < 0.05, Mann-Whitney test). **(B)** TEER values before (48h) and after (52h) exposure to the paracellular tracer CB or CB plus the different NLCs (CB, CB+TAT-CS-DiD, CB+CS-DiD). (Data are means ± SD of n=2-4 different Transwells, one differentiation.) **(C)** Orthogonal views from the different planes (x/y, x/z, or y/z) of confocal microscopy image (LSM 800, 100X objective, z-stack sectioning) showing the TAT-CS-DiD particle uptake. DiD (magenta) shows the different NLCs, ZO-1 tight junctions (orange), phalloidin the cell membrane (green) and DAPI the nuclei (blue). Scale bar 30 µm. (D) Confocal images of the hiPSC-derived BBB post-exposure to the different types of NLCs. DiD (magenta) shows the different NLCs, ZO-1 tight junctions (orange), Phalloidin the cell membrane (green) and DAPI the nuclei (blue). Image shows maximum projection of a z-stack (20 stacks). Scale bar 20µm. **(E)** Apparent permeability of the different mixes (CB, CB+TAT-CS-DiD, CB+CS-DiD) in the apical to basolateral direction across the

hiPSC-derived BMEC layer. (Data are means ± SD of n=3-4 different Transwells, one differentiation.)

Additional file 6

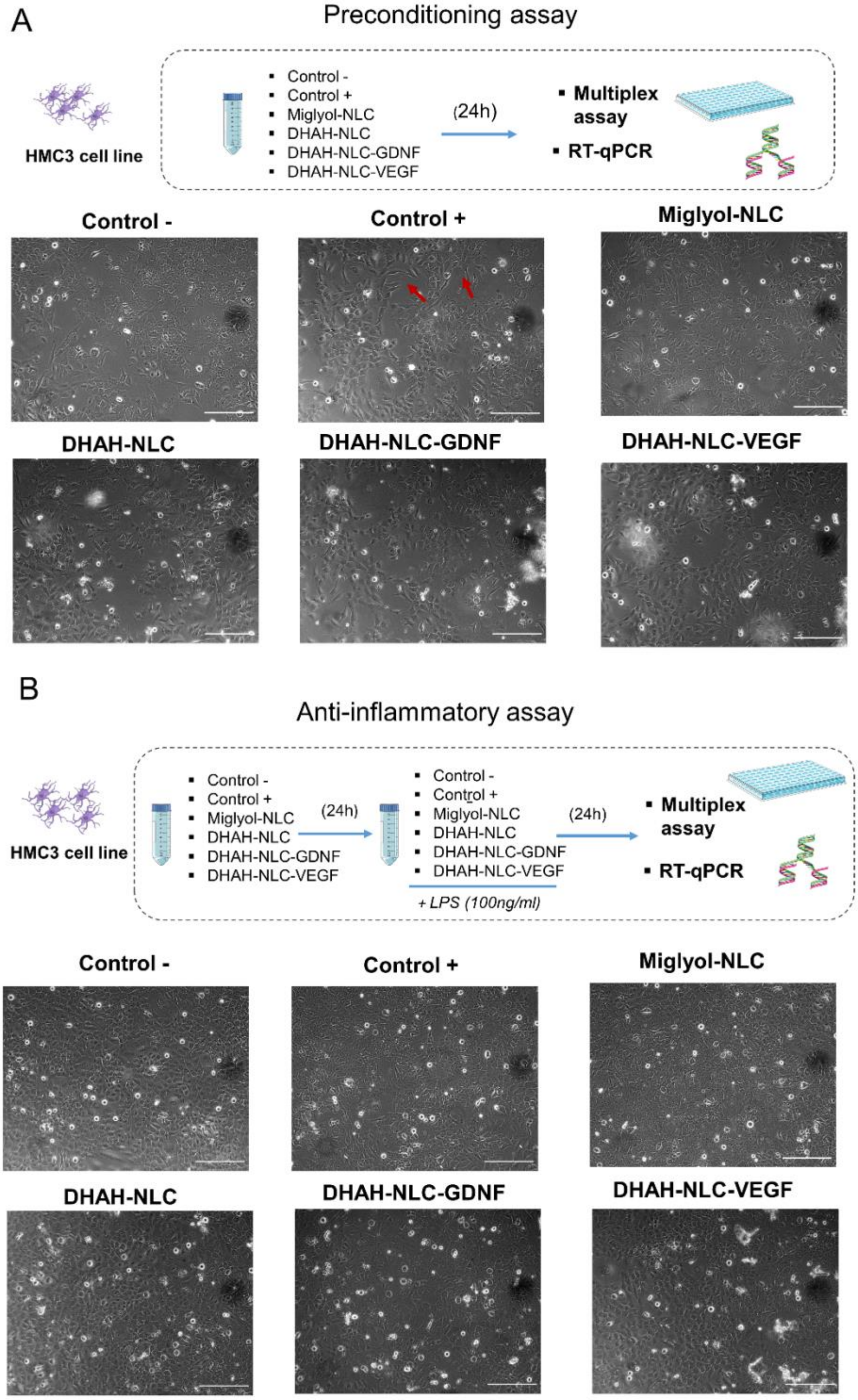

Additional file 6. NLC incubation -working concentrations set in AlamarBlue assay- in HMC3 microglia cell line (A) Schematic representation of the experimental procedure in microglial culture and bright-field images 24h after treatment with our NPs. Control $^{-}$ (media change) Control $^{+}$ (LPS 100 ng/ml) for preconditioning assay. The arrows indicate the slight change to amoeboid phenotype in microglia. Scale bar 200µM. **(B)** Schematic representation of the experiment conducted in HMC3 microglia and the obtained bright-field images at 48h. Control $^{-}$ (media change) Control $^{+}$ (LPS 100 ng/ml) for anti-inflammatory assay. Scale bar 200µM. This figure was created using Servier Medical Art templates, licensed under a Creative Commons Attribution 3.0 Unported License; *https://smart.servier.com*.

Additional file 7

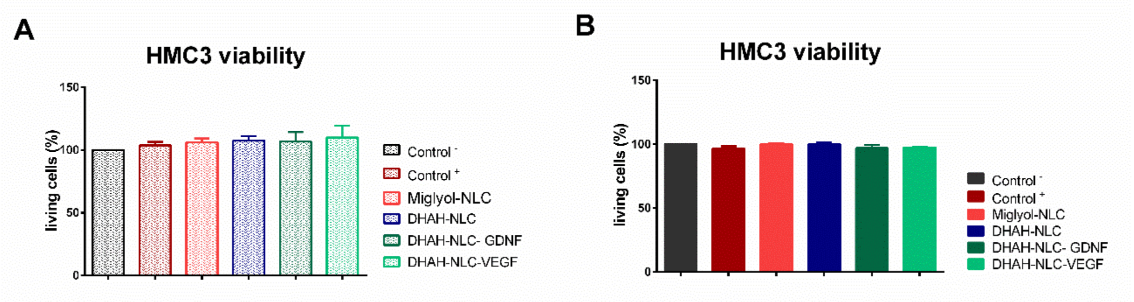

Additional file 7 HMC3 cell viability after exposure to LPS or NLC (Miglyol-NLC, DHAH-NLC, DHAH-NLC-GDNF, DHAH-NLC-VEGF in working concentrations). **(A)** Remaining living cells after exposure in preconditioning assay (Additional file 6). **(B)** Remaining living cells after exposure in anti-inflammatory assay (Additional file 6). Cell viability for each condition (treated cells) is expressed as the percentage of cell number compared to Control $^{-}$, which was set as 100%. No differences in cell viability were detected ($p>0.05$, One-way ANOVA). For both analyses, Control $^{-}$ denotes no treatment, media change and Control $^{+}$ denotes LPS incubation (100ng/ml).

Additional file 8

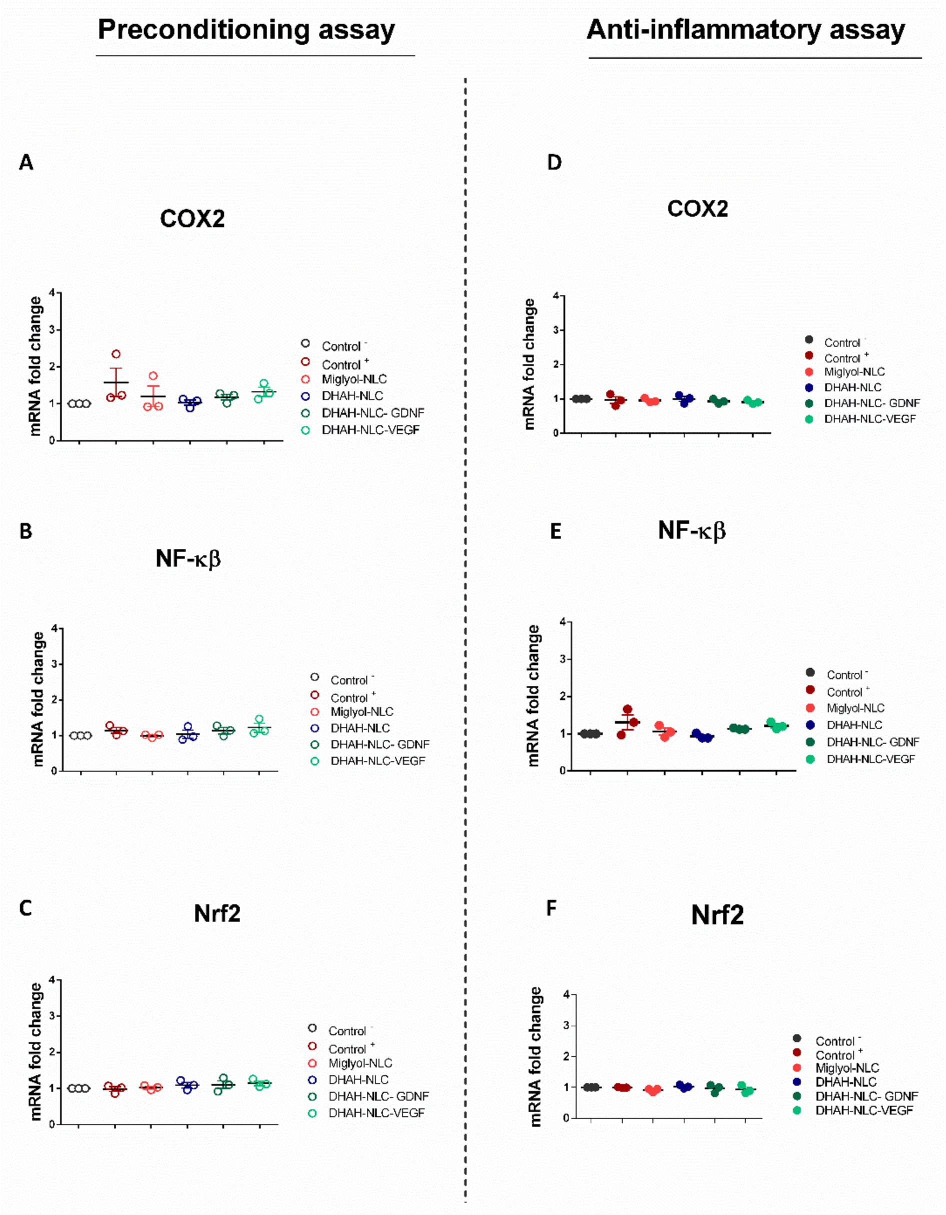

Additional file 8 Gene expression analysis (RT-qPCR). (A) COX2 **(B)** NF-κβ **(C)** Nrf2 for preconditioning assay (see also additional file 6). **(D)** COX2 **(E)** T NF-κβ **(F)** IL-1β and **(H)** Nrf2 for anti-inflammatory assay (see also additional file 6). Relative mRNA expression was normalized against GAPDH and, and the gene expression of the group that only media change was performed (Control^{-}) was used for normalization (value 1). (Data are presented means ±SEM of three independent experiments; ΔCT values were used for statistical analysis, One-Way ANOVA). In both conditions, Control^{-} denotes no treatment, media change and Control^{+} denotes LPS incubation (100ng/ml).

Additional file 9

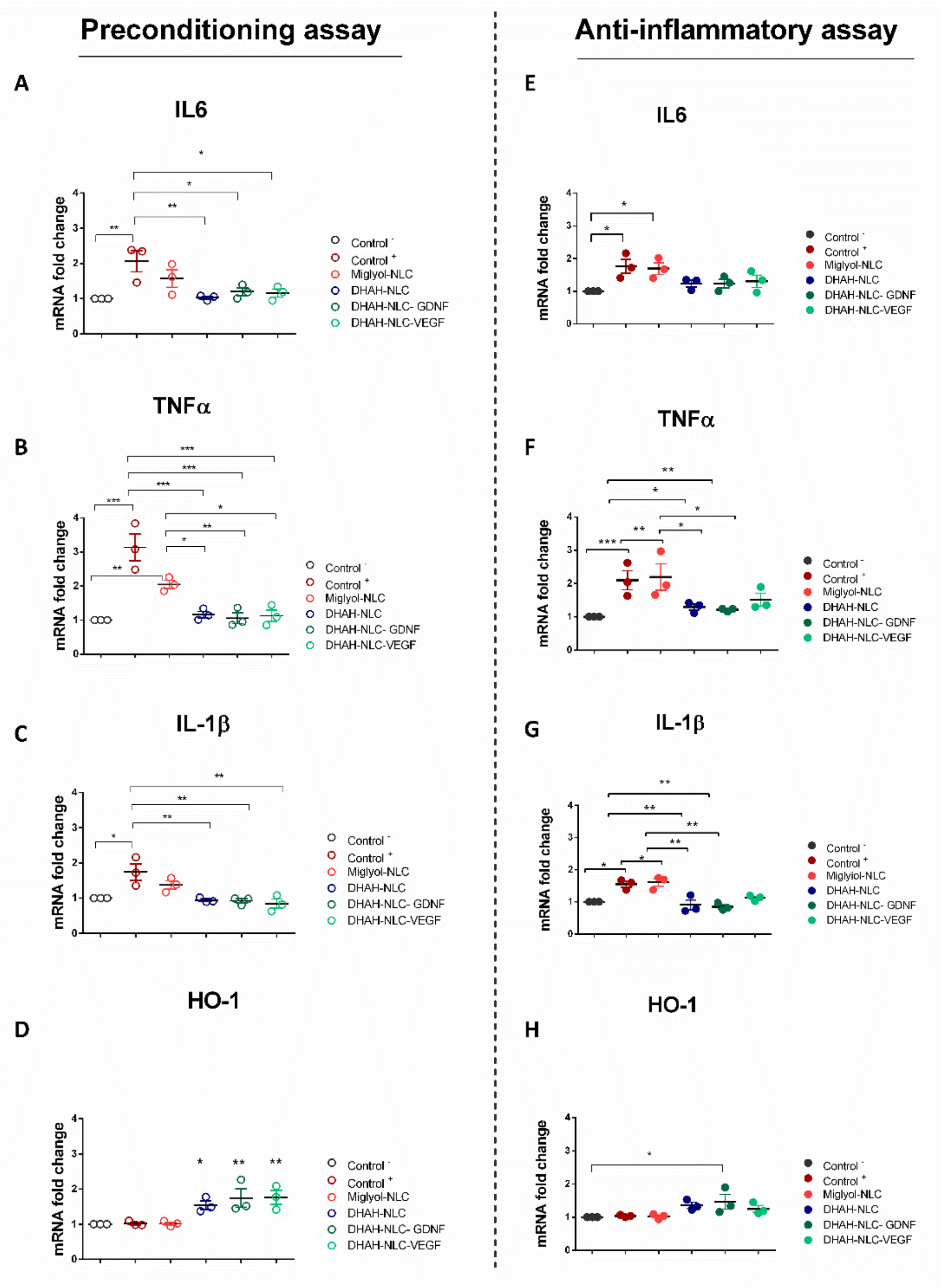

Additional file 9 Gene expression analysis (RT-qPCR). (A) IL6 **(B)** TNF-α **(C)** IL-1β and **(D)** HO-1 for preconditioning assay (Additional file 6). **(E)** IL6 **(F)** TNF-α **(G)** IL-1β and **(H)** HO-1 for anti-inflammatory assay (Additional file 6). Relative mRNA expression was normalized against GAPDH, and the gene expression of the group where only media change was performed (Control$^-$) was used as a reference (fold change 1). (Data are means ± SEM of three individual experiments; ΔCT values were used for statistical analysis, $^*p<0.05$ $^{**}p<0.01$ $^{***}p<0.001$. One-

Way ANOVA, Turkey's multiple comparison test. In both conditions, Control $^{-}$ denotes no treatment, media change and Control $^{+}$ denotes LPS incubation (100ng/ml).

Additional file 10

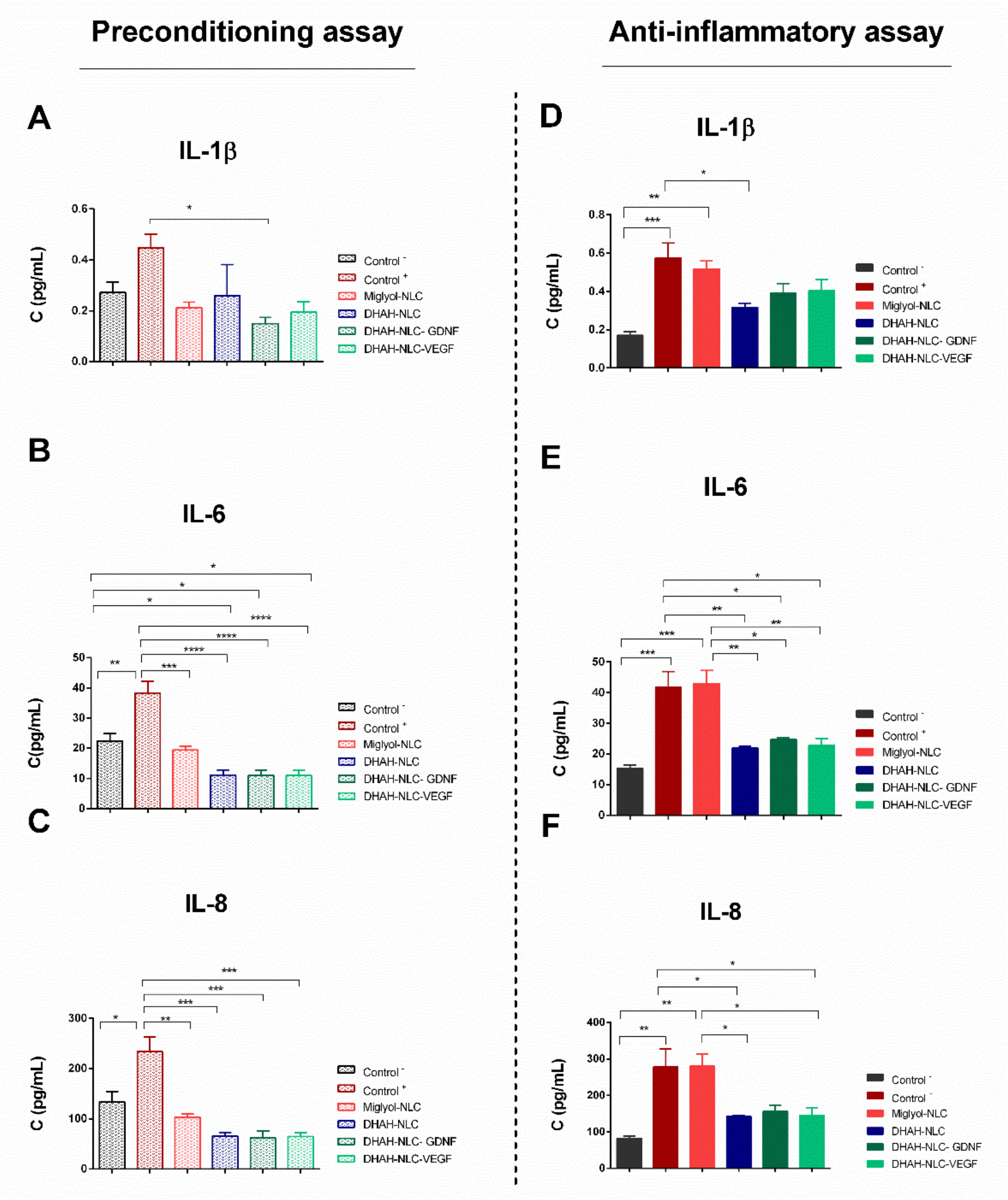

Additional file 10 Cytokine secretion analysis (U-PLEX Assay) assay values. **(A)** IL-1β **(B)** IL-6 and **(C)** IL-8 for preconditioning assay (Additional file 6). **(D)** IL-1β **(E)** IL-6 and **(F)** IL-8 for anti-inflammatory assay (Additional file 6). (Data are means ± SEM of three individual experiments. $^{*}p<0.05$ $^{**}p<0.01$ $^{***}p<0.001$, $^{****}p<0.0001$, One- Way ANOVA, Turkey's multiple comparison test). In both conditions, Control - denotes no treatment, media change and Control + denotes LPS incubation (100ng/ml).

Additional file 11

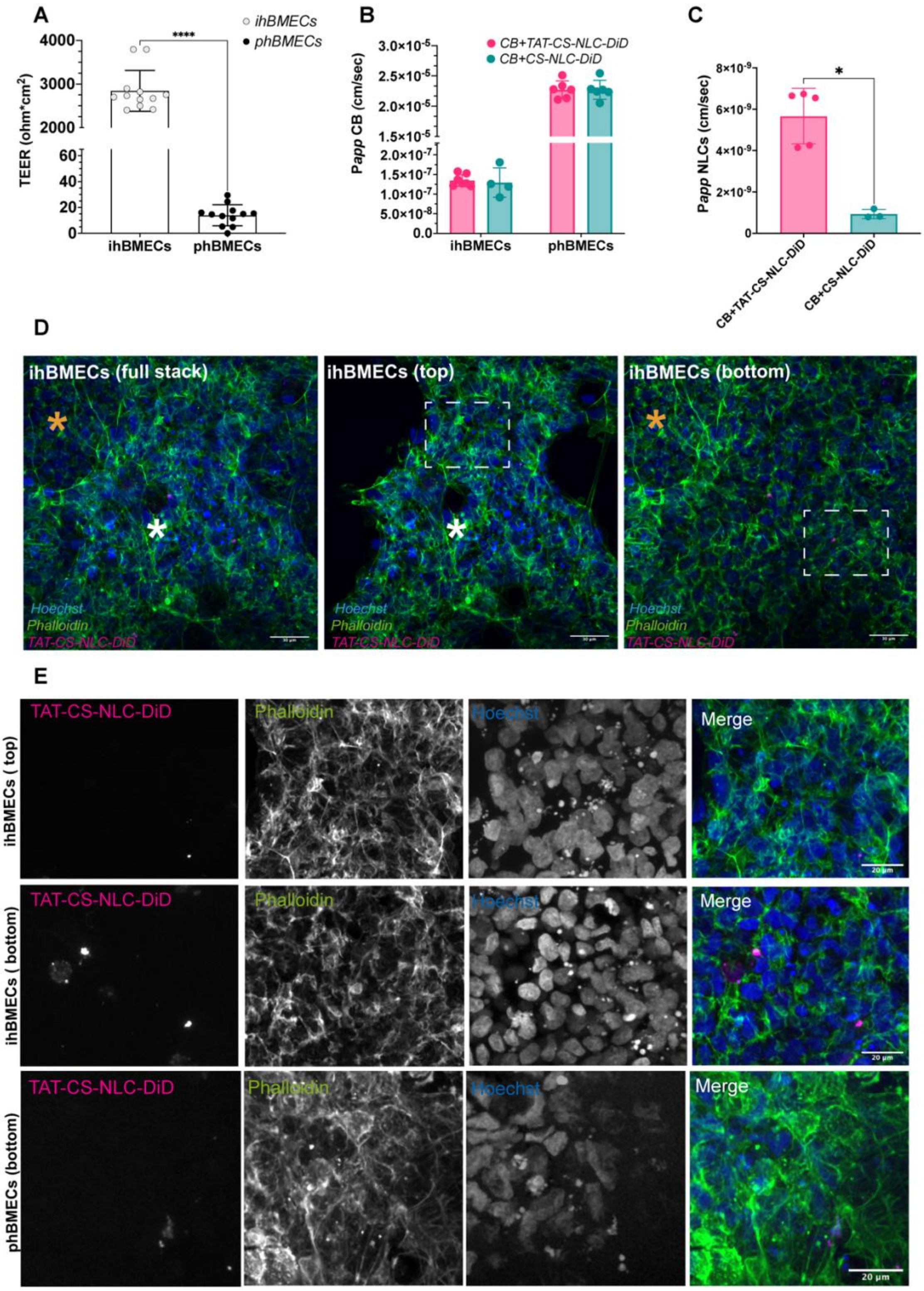

Additional file 11 NLC permeability across the human BBB.

(A) TEER values 48 post subculturing on Transwells for the hiPSC-derived BBB-like cells (ihBMECs) and for the primary human (phBMECs). (Data are means ± SD of n=12 Transwells,

one differentiation for hiPSC-BBB like cells, hBMECs p8, *** p<0.001, Mann-Whitney test). **(B)** Apparent permeability of CB in the apical to basolateral direction across the different BBB models. (Data are means ± SD of n=4-6 Transwells per treatment and cell type). **(C)** Apparent permeability of NLCs in the apical to basolateral direction across the ihBMECs. (Data are means ± SD of n=3-5 different Transwells per treatment.) **(D1-3)** Confocal images of the ihBMECs with entrapped particles. **(D1)** shows a maximum projection along the cell layer (63X, 80 stacks, 0.5 µM slicing, 40 µM range, Zoom 0.6). Asterisks indicate parts of the top (orange) and bottom (white) part of the layer (40 stacks, 0.5 µM slicing, 20 µM range, Zoom 0.6, top: upper part of the cell layer, bottom: lower side towards the membrane of the Transwell filter). DiD (magenta) shows the NLCs, phalloidin the cell membrane (green) and Hoechst the nuclei (blue). **(D2)** shows only the top part (20 µM range, white asterisk) and **(D3)** shows the bottom part (20 µM range). White boxes in (D2,3) indicate particle-containing areas that are shown in (E). **(E)** Zoom in the areas containing particles on the top and bottom part of the ihBMECs (two first rows) and on the bottom part of the phBMECs (third row). Scale bar 30 µm. Images were acquired with a Zeiss LSM800 confocal microscope using a 63x objective.

References

1. Matthiesen I, Voulgaris D, Nikolakopoulou P, Winkler TE, Herland A: **Continuous Monitoring Reveals Protective Effects of N-Acetylcysteine Amide on an Isogenic Microphysiological Model of the Neurovascular Unit.** Small 2021,:e2101785.

2. Stebbins MJ, Wilson HK, Canfield SG, Qian T, Palecek SP, Shusta EV: **Differentiation and characterization of human pluripotent stem cell-derived brain microvascular endothelial cells.** *Methods* 2016, **101:**93-102.
